# Flapping before Flight: High Resolution, Three-Dimensional Skeletal Kinematics of Wings and Legs during Avian Development

**DOI:** 10.1371/journal.pone.0153446

**Published:** 2016-04-21

**Authors:** Ashley M. Heers, David B. Baier, Brandon E. Jackson, Kenneth P. Dial

**Affiliations:** 1 Division of Paleontology, American Museum of Natural History, Central Park West and 79^th^ St., New York, New York 10024, United States of America; 2 Department of Biology, Providence College, 1 Cunningham Square, Providence, Rhode Island 02918, United States of America; 3 Biology and Environmental Sciences, Longwood University, 201 High St., Farmville, Virginia 23909, United States of America; 4 Division of Biological Sciences, University of Montana, 32 Campus Drive, Missoula, Montana 59812, United States of America; Brown University, UNITED STATES

## Abstract

Some of the greatest transformations in vertebrate history involve developmental and evolutionary origins of avian flight. Flight is the most power-demanding mode of locomotion, and volant adult birds have many anatomical features that presumably help meet these demands. However, juvenile birds, like the first winged dinosaurs, lack many hallmarks of advanced flight capacity. Instead of large wings they have small “protowings”, and instead of robust, interlocking forelimb skeletons their limbs are more gracile and their joints less constrained. Such traits are often thought to preclude extinct theropods from powered flight, yet young birds with similarly rudimentary anatomies flap-run up slopes and even briefly fly, thereby challenging longstanding ideas on skeletal and feather function in the theropod-avian lineage. Though skeletons and feathers are the common link between extinct and extant theropods and figure prominently in discussions on flight performance (extant birds) and flight origins (extinct theropods), skeletal inter-workings are hidden from view and their functional relationship with aerodynamically active wings is not known. For the first time, we use X-ray Reconstruction of Moving Morphology to visualize skeletal movement in developing birds, and explore how development of the avian flight apparatus corresponds with ontogenetic trajectories in skeletal kinematics, aerodynamic performance, and the locomotor transition from pre-flight flapping behaviors to full flight capacity. Our findings reveal that developing chukars (*Alectoris chukar*) with rudimentary flight apparatuses acquire an “avian” flight stroke early in ontogeny, initially by using their wings and legs cooperatively and, as they acquire flight capacity, counteracting ontogenetic increases in aerodynamic output with greater skeletal channelization. In conjunction with previous work, juvenile birds thereby demonstrate that the initial function of developing wings is to enhance leg performance, and that aerodynamically active, flapping wings might better be viewed as adaptations or exaptations for enhancing leg performance.

## Introduction

When we think about form and function in the vertebrate tree of life, we usually envision the adult phenotype. Whether an invasion of land during the origin of tetrapods or the acquisition of flight in a developing bird, vertebrate body plans are typically discussed with respect to the adult condition–particularly for transitions in locomotor mode. This “adultocentric” mindset [1] implicitly assumes that the adult body plan is most affected by selective forces, and that juvenile stages are simply a means to achieving adulthood.

Juvenile animals, however, add a unique and crucial perspective to form and function in the tree of life, by demonstrating how transitional, morphing anatomies function. Animals in many species must forage and find refuge at immature stages when they are underdeveloped and highly vulnerable, such that selection on juveniles for locomotor performance (precocial animals), or on parents for care that buffers young from the environment (altricial animals), is presumably intense [2–9]. Though sparsely studied, locomotor ontogeny is an integral element of understanding how species body plans are assembled along extant tips in the tree of life. In addition, many juveniles locomote using rudimentary morphological features that are not only missing the specializations of adult counterparts, but that also share similarities with “transitional” structures of extinct relatives [10]. Developing animals thereby illuminate deeper branching of the tree as well, by showing how transitions in form effect transitions in function and helping to elucidate the functional attributes of fossils with similar morphologies. Thus, though we know relatively little about juvenile locomotion and ecology, quantifying developmental transitions in form and function can provide important insight into biological patterns of both the present and past (for a discussion on Haeckel and the relationship between ontogeny and evolution, see Box 1 in [10]). This is true for many vertebrates [11–14], but particularly evident in developing birds.

Flight-capable birds are one of the most striking, textbook cases for form-function congruence [15]. Compared to other tetrapods and their theropod ancestors, extant adult birds have highly modified integuments and musculoskeletal systems, and it has long been assumed that many of these modifications are adaptations or exaptations (“aptations”) [16] for meeting the demands of flight. These ideas are so well accepted and ingrained in literature that avian flight is often discussed colloquially as a binary character: animals (or fossils) with “flight” aptations are flight-capable, animals without them are not. Developing birds, however, challenge this view.

**Fig 1:**
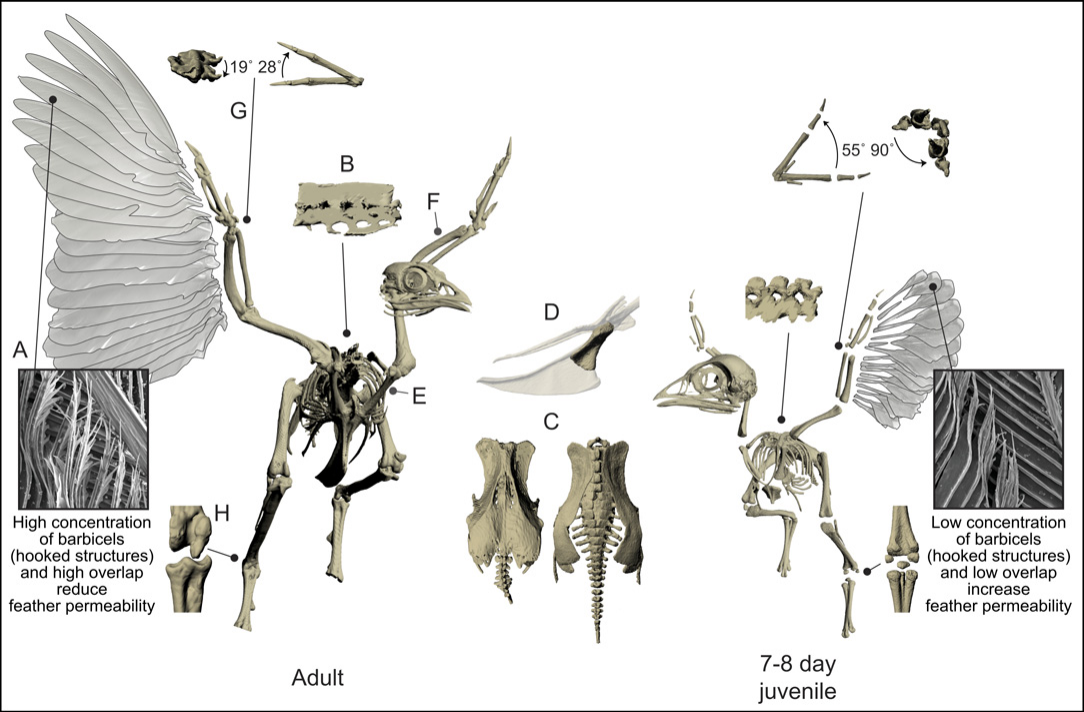
Ontogeny of flight aptations. **Left.** Flight-capable adult birds have many morphological features that are presumably adaptations or exaptations for meeting aerial challenges. Large wings with stiff, asymmetrical primary feathers (A) are thought to stabilize feathers against oncoming airflow [48], prevent excessive deformation [23], and reduce feather permeability [49]. Fused thoracic and sacral vertebrae may increase trunk rigidity and help transmit limb-generated forces to the rest of the body (notarium, B), and/or possibly act as a shock absorber during landing (synsacrum, C) [21,26,28,50]. Appendicularly, the robust forelimb apparatus (e.g., sternum with large keel (D), strut-like and well-articulated coracoids (E), bowed ulna (F)) allows for the attachment and contraction of powerful flight muscles (e.g., pectoralis, supracoracoideus) [27,28,26,25,21,51], while the acrocoracoid and triosseal canal (not shown; present in juveniles but less elevated above glenoid) allow the supracoracoideus muscle to act as a pulley, contributing to humeral elevation and rotation [28,26,29–33]. Distally, reduced and fused skeletal elements and channelized limb joints (G, H) are thought to reduce mass and permit rapid, efficient limb oscillation, coordinate elbow and wrist movement, keep a planar wing orientation during the downstroke, and increase stride effectiveness by restricting ankle movements to a single plane of motion [27,28,26,22,52]. Collectively, these features are a key component of the avian *bauplan*, and a classic example of anatomical specialization. **Right.** Developing birds–like early winged dinosaurs–lack many hallmarks of advanced flight capacity [10]. Instead of large wings they have small protowings, with a more gracile skeleton and less constrained joints. Immature birds nevertheless flap their rudimentary wings to accomplish a variety of locomotor tasks [20,53–55]; in fact, many anatomical specializations of adults are acquired long after flight capacity is achieved. Developing birds thereby challenge the traditional, longstanding view of form-function relationships in the theropod-avian lineage (A-H). Cervical vertebrae and pedal phalanges not shown; juvenile keel on top of adult keel, for scale (D); in (G), left image is pronation of carpometacarpus, right is abduction (juvenile joints always more flexible). Although cartilaginous skeletal components are not shown, this does not alter functional interpretations of the juvenile skeleton (e.g., juveniles possess a small cartilaginous extension of the keel, but both the keel and the muscles that attach to it are still proportionally much smaller in juveniles than adults; carpal bones of developing birds have the specialized shapes of adults, but are poorly ossified and not capable of resisting enough joint torque to channelize the wrist joint (G)). Images of feather microstructure (A) reprinted from [18] under a CC BY license, with permission from The Company of Biologists Limited, original copyright 2011.

Like early winged theropods (non-avian pennaraptorans and basal avialans), immature birds lack many hallmarks of advanced flight capability. Instead of large wings they have small “protowings”, and instead of hypertrophied pectoral muscles and a robust, interlocking skeleton, their musculoskeletal apparatus is more gracile and their joints less constrained [10,17–20] (Fig 1). Such features are often assumed to preclude avian ancestors from powerful flight and bird-like wingstrokes [21–23], because it is generally thought that:

- Protowings and small amounts of aerodynamic force production may help slow aerial descents, but otherwise do not to contribute to locomotion (i.e., what use is half a wing? [24])
- Small or nonexistent keels, short coracoids, and a lack of robust skeletal processes are indicative of small or weak flight muscles, and would result in a “reduced” wingstroke (reduced stroke amplitude and/or wingbeat frequency) [21,25–28]
- Lack of an expanded acrocoracoid and/or triosseal canal would reduce the ability of the supracoracoideus muscle to elevate and rotate the humerus during the upstroke [28,26,29–33]
- Relatively unconstrained elbow and wrist joints would result in (i) less coordinated elbow and wrist movements [22,28], and (ii) detrimental wing deformation, because wing joints would not be able to resist the aerodynamic forces that pull up on a wing during flight, and would abduct (bend) or pronate (twist) into less aerodynamically effective orientations [27,28,26,22]
- A relatively small flight apparatus suggests a more posterior center of mass, which should affect hind limb kinematics and might result in a less crouched posture to maintain balance [34–36]

These ideas are intuitive, consistent with the morphologies of adult birds, and often extrapolated to infer that animals with small wings and relatively gracile, unchannelized forelimb skeletons either do not use their feathered forelimbs at all (developing birds), or used them passively (e.g., gliding) and/or for non-locomotor purposes, such as display (many extinct theropods) (Table 1 in [10]; [37–47]).

Developing birds challenge this adultocentric view because fledglings with very rudimentary anatomies begin flapping and producing aerodynamic forces long before acquiring “flight” aptations and the “avian” body plan characteristic of adults [10,18,56]. Juveniles in species with a diverse array of wing-leg morphologies and life history strategies frequently recruit their legs and incipient wings cooperatively to avoid predators or reach refuges ([57–59]; AMH, KPD, and Tom Martin, personal observations; see juvenile birds in: https://www.youtube.com/watch?v=k94EDd8aKng and https://www.youtube.com/watch?v=3USAC-Ky25s), by flap-running up slopes (wing-assisted incline running / walking; WAIR) and controlling aerial descents [53,54], swimming and steaming across water [55], and/or jumping into brief flapping flights [20]. In fact, in at least some precocial species, the development of flight capacity (~18–20 days in *Alectoris chukar*) precedes the development of an adult-like musculoskeletal apparatus (close to ~100 days) by a substantial and biologically relevant margin [10,20]. Immature birds thereby call into question many longstanding views on form-function relationships in the avian body plan: why are these animals capable of aerodynamically active flapping behaviors in the apparent absence of many “flight” aptations?

Here, we integrate a series of previous studies on Chukar Partridges (*Alectoris chukar*; precocial ground bird) with X-ray Reconstruction of Moving Morphology (XROMM; www.xromm.org), to explore how the development of “flight” aptations (Fig 1) corresponds with ontogenetic trajectories in skeletal kinematics, aerodynamic performance [18,56], and the locomotor transition from pre-flight flapping behaviors (e.g., WAIR, steaming) to full flight capacity [57,53–55,20,60,61]. Flapping kinematics are integral to the ability to fly and figure prominently in discussions on flight performance (neontology) and the origin of flight (paleontology), yet skeletal inter-workings are hidden from view and their functional relationship with aerodynamically active wings is not known. To examine this relationship and better understand how fledglings with rudimentary forelimb apparatuses generate useful aerodynamic forces as they acquire flight capacity, we used XROMM to quantify ontogenetic trajectories in three-dimensional wing and leg kinematics during a pre-flight flapping behavior–WAIR–and test three hypotheses:

- (H1) From an adultocentric perspective, the rudimentary flight apparatus of developing birds should hamper locomotor performance by forcing juveniles to flap with different (non-bird-like) kinematics. Alternatively,
- (H2) kinematic differences could occur during WAIR due to differences in wing length, with the longer wings of older birds being inhibited by contact with the substrate, or
- (H3) kinematic differences could occur due to different levels of effort, since juveniles are inherently weaker and should struggle more to ascend steep obstacles.

A lack of kinematic differences would suggest some sort of compensatory mechanism in younger birds. We discriminated between these possibilities by comparing skeletal kinematics for four ontogenetic stages (7–8 days post-hatching (dph), 11–12 dph, 18 dph, adult) flap-running on shallow substrate angles (60–65°), and for two ontogenetic stages (23 dph, adults) flap-running on different substrate widths (narrow versus wide) or on steeper substrate angles (70–80°) (Table A in S1 File).

**Fig 2:**
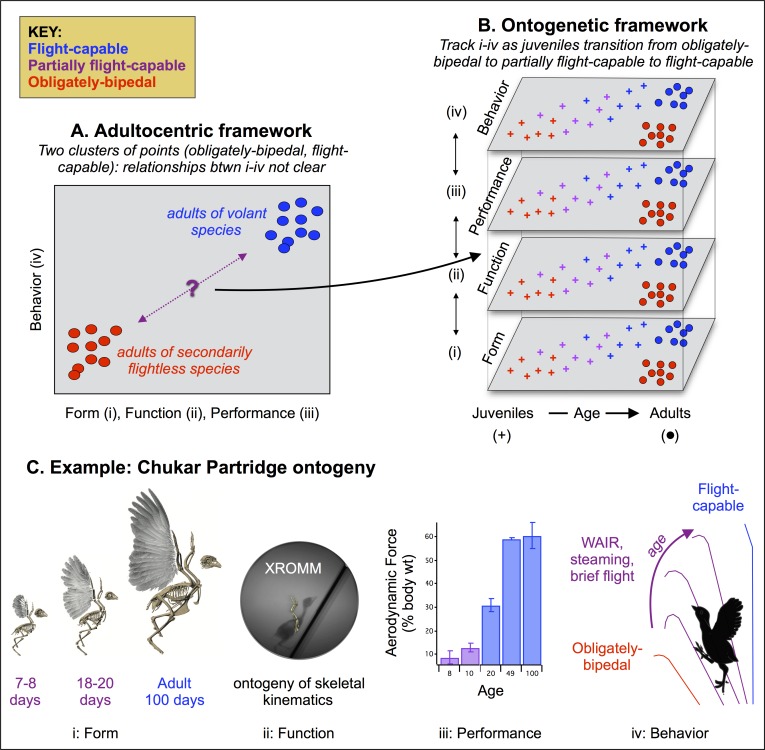
Relationships between form, function, performance, and behavior. Locomotor ontogeny provides key functional, ecological, and evolutionary insight into the avian body plan, by revealing how transitional, morphing anatomies function. (A) From a functional perspective, adult birds are the endpoints of an ontogenetic and evolutionary continuum and cannot clearly elucidate how specific morphological attributes affect to the ability to become airborne. Nearly all adult birds share a suite of specialized morphologies, are flight-capable or secondarily flightless, and may not provide enough variation in morphology and flight capacity to expose relationships between these two variables (though adult birds of some species fly poorly [62], they have not been studied). In a traditional, adultocentric framework that perceives many morphological features as aptations for aerial locomotion, most relationships between form and locomotor function are therefore assumed rather than empirically tested. (B) Juvenile birds have rudimentary locomotor structures, and engage in pre-flight flapping behaviors as they morph into adulthood and acquire flight capacity. Though poorly studied [7,57], morphing juveniles fill a longstanding gap in knowledge and help clarify functional attributes of the avian body plan. By revealing form-function relationships that underlie obligately-bipedal to flight-capable transitions (i-iv), and thereby establishing how features are related to flight, developing birds can provide key insight into locomotor aptations. (C) For example, previous work has shown that juvenile chukars with rudimentary flight apparatuses (image i) transition from leg- to wing-based modes of locomotion by using their legs and wings cooperatively, and generating small but important amounts of aerodynamic force (iii, data from [18]) that increase throughout ontogeny and allow birds to flap-run up steeper obstacles and eventually fly (iv, data from [20,53,55,61]). Here, we quantify the ontogeny of skeletal kinematics (ii), to better understand relationships between form, function, performance, and behavior. Image i in (C) reprinted from [10] under a CC BY license, with permission from Cell Press, original copyright 2012.

Collectively, these treatments build upon previous work to investigate musculoskeletal-kinematic-aerodynamic relationships, and offer a more holistic perspective on form-function relationships in the avian *bauplan* (Fig 2). In conjunction with previous studies, our results help clarify the functional interplay between rudimentary musculoskeletal apparatuses and aerodynamically active, flapping protowings / wings, and thereby offer important insight into both the ontogenetic and evolutionary acquisition of flight capacity.

## Materials and Methods

### Animals, age classes, and treatments

Chukars were purchased from commercial breeders as day old chicks or as adults over the course of two breeding seasons (2009, 2011). All birds were fed *ad libitum* in temperature-controlled rooms (chicks) or in outdoor aviaries (adults), either at the Field Research Station at Fort Missoula, University of Montana, MT, USA, or at the Brown Animal Care Facility, Brown University, RI, USA. The University of Montana and/or Brown University Institutional Animal Care and Use Committees approved all protocols. A few days prior to collecting x-ray videos, we implanted 0.5–1 mm tantalum bead markers in the left humerus, ulna, radius, and carpometacarpus of one anesthetized adult and two anesthetized immature birds (marker-based XROMM [63], 2011), at the Field Research Station; we did not implant markers in 2009 (markerless XROMM / scientific rotoscoping [64]) due to the small size and extensive cartilaginous components of juvenile bird bones.

We chose four age classes for analysis, based on ontogenetic transitions in morphology and locomotor capacity (Table A in S1 File). For each bird in each age class, we used markerless and/or marker-based XROMM to animate at least one full wingstroke (left and right limbs for juveniles, left limb for adults due to size constraints) and one full stride cycle (one limb in stance and the other in swing) during WAIR, focusing on the three major joints in the forelimb and hind limb (shoulder, elbow, wrist, hip, knee, and ankle). Each wingstroke and stride cycle analyzed was from one continuous trial (no partial strokes or cycles).

All birds were filmed flap-running on ramps angled at 60–65° (Treatment 1) (n = 2 individuals for adults, data from [60]; n = 3 individuals for juveniles due to higher expected variability and/or difficulty in rotoscoping) (Table A in S1 File). To distinguish between our three alternative hypotheses (H1-H3), we additionally filmed older juveniles and adults at different ramp widths (Treatment 2) (n = 2 individuals) or at steeper angles (Treatment 3) (n = 1 individual, due to experimental constraints). For treatments with smaller sample sizes (n = 1–2 individuals), we animated multiple wingbeats and stride cycles per bird (all from separate trials). Any trials in which the bird tripped, jumped, or paused were discarded.

### X-ray video collection

We filmed all trials in the W. M. Keck Foundation XROMM Facility at Brown University. Birds performed WAIR on medium grit, sandpaper-covered ramps centered in the beam field of two Varian model G-1086 x-ray tubes (40 kV, 200–250 mA, magnification 0–2; continuous radiation) and two Dunlee model TH9447QXH590 image intensifiers (SID ~90 cm) (S1 Video; www.xromm.org). For trials at 60–65° (Treatments 1, 2), we positioned the x-ray tubes dorsally and laterally to the birds. For trials at steeper angles (Treatment 3), we positioned the x-ray tubes ventrolaterally and dorsolaterally. X-ray video outputs of the image intensifiers were captured either by two synchronized high-speed 1200x1200 Phantom v10 digital video cameras recording at 500 fps and shutter speeds of 1/400-1/1000 s (all data except Treatment 1 adults), or by two 1024x1024 Photron cameras (500 fps, shutter speeds of 1/6000 s; Treatment 1 adults [60]).

### Video calibration and undistortion

We collected images of a perforated metal grid and calibration cube images prior to and after each day of video collection to remove distortion and calibrate the cameras [63].

### CT scans and skeletal models

After filming each age class, we euthanized and froze all birds. We then scanned individuals at the Rhode Island Hospital (CT; 80 kV, 400 mA, 0.625 mm slice thickness) (Treatment 1 adults), at the University of Texas at Austin (www.digimorph.org) (CT; 190–200 kV, 0.17 mA, 0.07313–0.09751 mm slice thickness) (Treatment 1 juveniles), or at the Digital Imaging Facility at Harvard University (CT or microCT; 116–152 kV, 0.043–0.068 mA, 0.0749–0.1075 mm slice thickness) (Treatments 2 and 3). Skeletal elements and markers, if present, were segmented out in Amira 4.0 (Mercury) or in OsiriX v.4.0 32-bit, and saved as polygonal mesh models. Inertial axes were calculated for each mesh model, using MATLAB [65].

#### Scientific rotoscoping / markerless XROMM (all juveniles, adult hind limbs)

Mesh models and their respective axes were imported into Maya (Autodesk) and used to create an anatomical reference pose and a hierarchical “puppet” [64] with a joint coordinate system [66] allowing each individual bone to be rotated and/or translated about its respective joint. Joint coordinate systems for the pelvis (whole body motion) and sternal, coracosternal, shoulder, elbow, wrist, hip, knee, and ankle joints were defined using inertial axes and anatomical landmarks, as in [60], such that each joint was allowed 6 degrees of freedom (3 translational, 3 rotational) and rotation occurred first around the z-axis, then y-axis, then x-axis (xyz rotation order in Maya) (Figure A in S1 File). Movement about a limb joint (e.g., shoulder joint) resulted in movement of the distal bone defining the joint (e.g., humerus), as well as movement of all downstream elements (elbow joint, ulna, radius, wrist joint, carpometacarpus), such that translation and rotation values changed only at the joint being manipulated and were defined as movement of the distal bony element with respect to its more proximal limb element. Bird morphology at a given juvenile stage was similar enough that we constructed only one skeletal model for each juvenile age class; for adults, we constructed a model for each individual (see [60]).

#### Marker-based XROMM (adult forelimbs)

Here, joint coordinate systems were established in the same way as markerless Rotoscoping models (Figure A in S1 File), but bone position and orientation were driven by digitized markers. Joint movements were then calculated from the joint coordinate system using the “joint axes tool” in the XROMM tools package for Maya (www.xromm.org).

### Animation

#### Scientific rotoscoping / markerless XROMM (all juveniles, adult hind limbs) (S2 Video)

Following [64] and [60], we used the joint coordinate system of the hierarchical puppet to manually translate and rotate each bone into proper position and orientation in both x-ray views. Each bird was first positioned and oriented by aligning the thoracic vertebrae and pectoral and pelvic girdles to both x-ray views. Individual limb bones were then aligned, starting with the more proximal elements and working distally (downstream). We were unable to accurately rotoscope long axis rotation of the tibiotarsus or tarsometatarsus in the youngest juveniles. In order to compare kinematics across age classes, we therefore assumed that no long axis rotation or abduction/adduction occurred at the ankle, and rotoscoped the knee accordingly (consistent with this assumption, the ankle joint of guineafowl (*Numida meleagris*) closely resembles a hinge joint with one degree of freedom [67]).

#### Marker-based XROMM (adult forelimbs)

Following [63], we used a customized script within MATLAB [68] to digitize the position of each bone marker in the undistorted and calibrated x-ray views, for every frame of every trial. Digitized marker positions were saved as three-dimensional xyz points. For the humerus and carpometacarpus (3 markers each), we used the “rigid body motion tool” in the XROMM tools package for Matlab (www.xromm.org) to combine these digitized marker positions with centroid positions, calculate the three-dimensional position and orientation of the bone, and import the position and orientation data into Maya. For the ulna and radius (1 marker each), we similarly imported the position data into Maya, but then manually rotated each bone about its marker until properly oriented in both x-ray views. Once all bones were properly aligned, we used the “joint axes tool” in the XROMM tools package for Maya to measure rotations and translations of each limb bone about the limb element more proximal to it.

### Wingstroke and stride cycle transitions

Following [60], we defined the upstroke-downstroke transition as the point in the wingstroke cycle when the tip of the manus reaches its maximum distance above the sternum (dorsoventral distance in a transverse section centered at, and angled perpendicular to, the sternum; distance independent of body orientation). Conversely, we defined the downstroke-upstroke transition as the point in the wingstroke cycle when the tip of the manus reaches its minimum dorsoventral distance above the sternum. For the legs, we identified transitions between stance and swing phase visually: the beginning and end of stance were defined as toe-down and toe-off, respectively, and swing was defined as the aerial phase between toe-off and toe-down.

### Data analysis

Once we had animated each trial and identified phase transitions, we imported raw translations and rotations of each joint into IGOR PRO v6.12 (Wavemetrics Inc) for data preparation and visualization, and into R [69] for statistical analyses. In order to statistically compare different birds and different trials, we first scaled the raw data (all limbs, all trials; IGOR PRO) such that each wingstroke or stride cycle was 101 frames in length, with the duration of the downstroke or stance phase representing 50% of the duration of the stroke cycle or stride cycle, respectively (1 frame for downstroke-upstroke and stance-swing transitions). To visually compare mean kinematics across age classes, for each kinematic rotation (x, y, z) at a given joint (shoulder, hip, etc.) we calculated the mean rotation of a given age class and treatment, at each point of the stroke or stride cycle. Averages were then smoothed using a Loess function (IGRO PRO), to adjust for kinematic discontinuities (some birds started in downstroke / stance, others started in upstroke / swing).

#### Treatment 1

To identify ontogenetic patterns, for each kinematic rotation at a given joint we calculated the mean, maximum and minimum values, and range of motion (maximum–minimum) of the entire stroke or stride cycle, for each trial (no filtering; average values of left and right wing used for juveniles). Patterns can occur as (i) ontogenetic trends, where a kinematic parameter either increases or decreases throughout ontogeny, and/or (ii) differences between adults and juveniles, where the adult values are either greater or less than those of all juveniles collectively. To identify these two types of patterns, we used R to calculate Spearman’s rank correlation coefficients (cor.test function for kinematic parameter versus age; ontogenetic trends) and run two sample two-sided Welch’s t-tests (t.test function for adults versus all juveniles; ontogenetic differences) on each kinematic parameter (mean, maximum, minimum, range) of each kinematic rotation (x, y, z). Downstroke and upstroke were analyzed together for movements with one maximum and one minimum (e.g., elevation and depression at shoulder, flexion and extension at wrist), and separately for movements with multiple maxima and minima (e.g., long axis rotation at elbow and wrist); however, if downstroke and upstroke showed the same statistical patterns, we re-ran the statistical analyses and analyzed downstroke and upstroke together. We analyzed stance and swing similarly. Given that some of the individuals were measured at two different ages or during two different trials (Table A in S1 File; supplemental results), we also ran linear mixed effects models (to assess ontogenetic trends; lme function in R) and calculated Tukey Contrasts (to compare adults versus all juveniles; lme and glht functions in nlme and multcomp packages) on kinematic parameters for each joint, using age rank as a fixed factor and bird identity as a random factor. We used age rank rather than actual ages because we were unsure of adult ages and at what point in ontogeny adult kinematics are acquired. Given that correlation coefficients and t-tests ignoring bird identity use data points that are not entirely independent of one another, but that mixed effects models with only two (hind limbs) or four (forelimbs) repeated measures lose degrees of freedom without offering improvement over OLS models (average log likelihood test p-value = 0.84), we report the results of both efforts here in an attempt to bracket p-values of kinematic parameters.

#### Treatment 2

To establish whether ramp width influences joint kinematics and wing depression, we measured wing kinematics and dorsoventral distances (distance in a transverse section centered at the sternum; distance independent of body orientation) between the tip of the manus and the center of the keel and the tip of the manus and the substrate, for two 23 day old birds flap-running on both narrow and wide ramps (multiple wingbeats per bird) (Table A in S1 File). We then compared minimum dorsoventral distances (expressed as a percentage of trunk length) for the two ramp widths using two sample one-sided Welch’s t-tests and Tukey Contrasts, as in Treatments 1 and 3. Relationships between kinematic values (x, y, z rotations) and minimum dorsoventral distances were plotted and visually assessed. We did not examine hind limb kinematics on different ramp widths because birds do not appear to coordinate their wing and leg movements during WAIR, so hind limb kinematics are random with respect to the downstroke-upstroke transition and minimum dorsoventral distance.

#### Treatment 3

Finally, to determine whether level of effort impacts joint kinematics, we compared adults flap-running at shallow (65°) and steep (70–80°) angles (Table A in S1 File). For each kinematic parameter (mean, maximum, minimum, range) of each joint rotation (x, y, z), we used two sample one-sided Welch’s t-tests and one-sided Tukey Contrasts (see Treatment 1) to determine whether adults flap-running at steep angles are more kinematically similar to juveniles than are adults flapping at shallow angles (i.e., whether juveniles flap-running at shallow angles and adults flap-running at steep angles are less different than juveniles and adults both flap-running at shallow angles).

### Validation

To assess how accurately we were able to rotoscope flap-running birds, we compared marker-based and rotoscoped joint rotations at the shoulder, elbow, and wrist, for one adult and one juvenile (9 dph) bird. Given that forelimbs are more challenging to animate than hind limbs during WAIR (higher rates of oscillation, less ossified in juveniles), they provide a conservative estimate of rotoscoping accuracy.

For the adult bird, we implanted markers into the humerus, ulna, and carpometacarpus prior to euthanasia, filmed the bird flap-running, then euthanized the bird and calculated marker-based joint rotations as described above (see “CT scans and skeletal models”, “Animation”). To check rotoscoping accuracy and precision, we measured the same sequence of video once using markers, and once via rotoscoping after digitally removing marker shadows from the x-ray images using the clone stamp tool in Adobe Photoshop. D. Baier removed the markers from the video, and A. Heers (who rotoscoped all juveniles, and adults on steep angles) manually rotoscoped the bones over the same set of frames (see scientific rotoscoping under “Animation”, above).

For the juvenile bird, since we were not able to implant markers on the live bird and compare techniques over a wingbeat during WAIR, we compared markerless and marker-based XROMM by elevating and depressing the wing postmortem. To do this, we implanted 3–4 markers on the sternum, humerus, ulna, and carpometacarpus, then filmed the bird “raising” and “lowering” its wing by securing the bird with a wooden dowel and pulling up on its primary feathers. We determined markerless and marker-based joint rotations of the shoulder, elbow, and wrist the same way as for the adult, with two exceptions. (E1) For rotoscoping the humerus, we had to give the shoulder joint the same positions as the shoulder axes in the marker-based file, because in contrast to our original data the proximal end of the humerus was nearly completely obscured by the dowel used to secure the bird; humerus orientations were still rotoscoped manually. (E2) For the marker-based positions and orientations of the sternum (and downstream coracoids), we used the position defined by the markers but determined the orientation by rotoscoping, because collinear markers along the sternal keel prevented accurate measure of sternal long-axis rotation.

As expected, rotoscoping accuracy varies slightly depending on bone identity and bird age, but margins of error are small and would not override our results (see Figure B in S1 File for full discussion).

## Results

Developing and adult birds flap-running on 60–65° inclines display a number of kinematic differences, expressed either as ontogenetic trends (7–8 dph → 11–12 dph → 18 dph → adults) or as collective disparities between juveniles and adults (all juveniles versus adults) (H1). These differences are not due to the length of the wings with respect to substrate width (H2), and largely disappear when birds are compared at similar levels of effort (H3). Thus, immature and adult birds with very different skeletal morphologies (Fig 1) are nevertheless capable of performing very similar skeletal movements, and in spite of lacking many “flight” aptations, developing birds implement adult-like flapping kinematics.

### H1: all birds on shallow (60–65°) inclines

**Fig 3:**
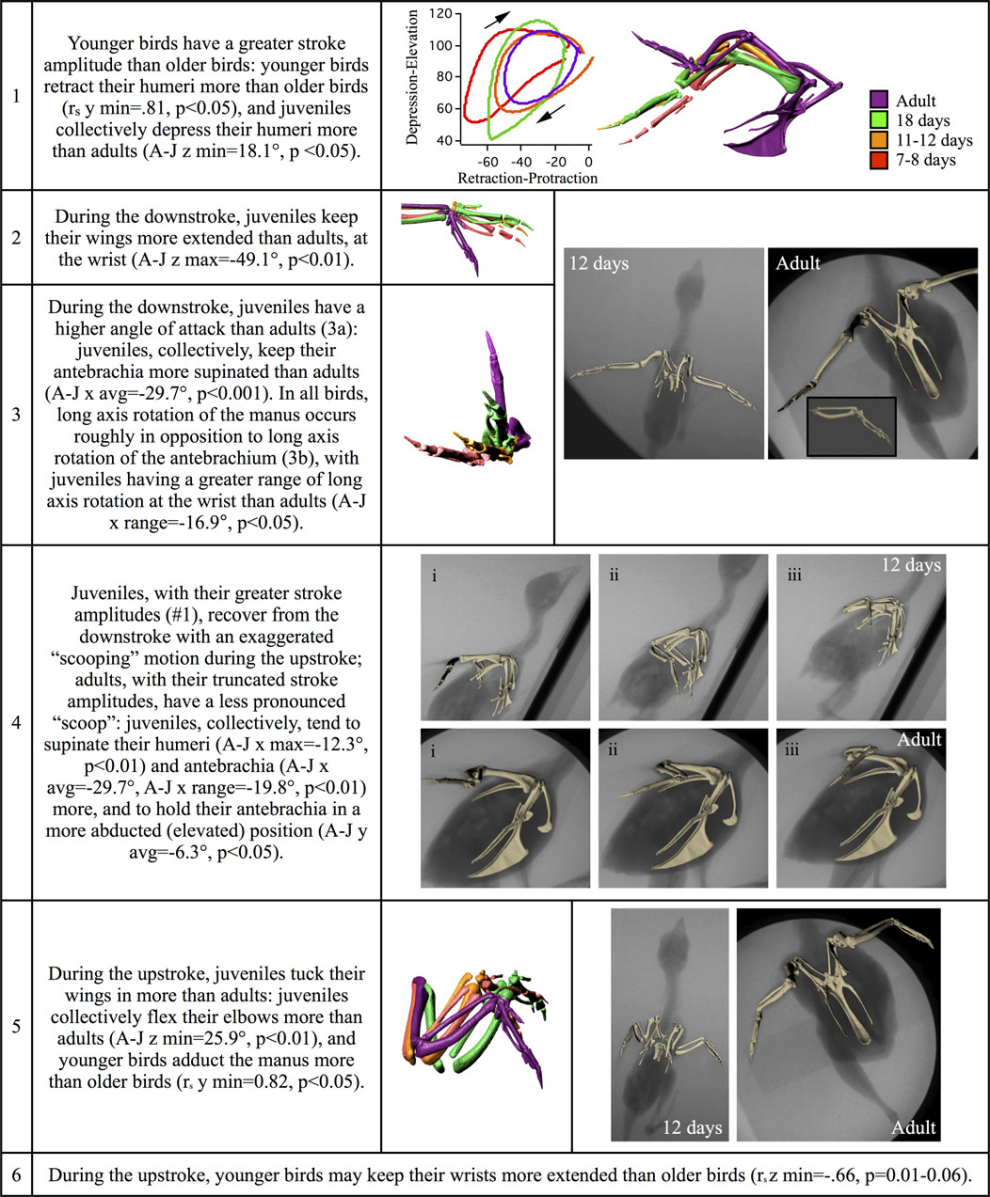
Ontogenetic trends and differences between adults and juveniles flap-running up 60–65° inclines: forelimb kinematics. r_s_: Spearman’s rank correlation coefficient; A-J: adult mean–juvenile mean; x, y, z: joint rotations shown in Fig C (z, top row: elevation-depression (shoulder) or flexion-extension (elbow, wrist); y, middle row: protraction-retraction (shoulder) or abduction-adduction (elbow, wrist); x, bottom row: long axis rotation); avg (average), max (maximum), min (minimum), and range: kinematic variables tested for statistical significance (Table E in S1 File).

**Fig 4:**
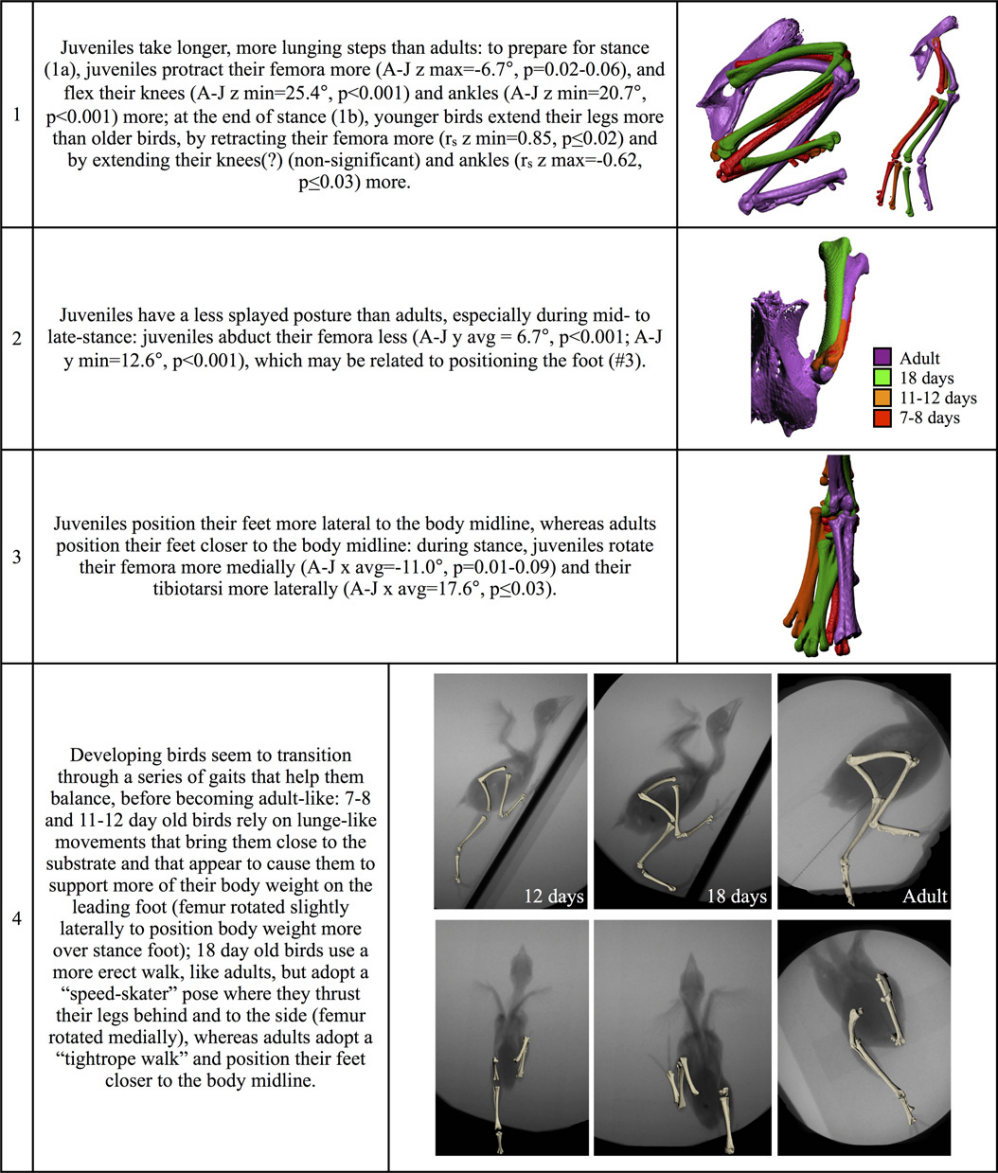
Ontogenetic trends and differences between adults and juveniles flap-running up 60–65° inclines: hind limb kinematics. r_s_: Spearman’s rank correlation coefficient; A-J: adult mean–juvenile mean; x, y, z: joint rotations shown in Fig D (z, top row: flexion-extension; y, middle row: abduction-adduction; x, bottom row: long axis rotation); avg (average), max (maximum), min (minimum), and range: kinematic variables tested for statistical significance (Table E in S1 File).

In general, juvenile and adult chukars produce similar types of movement (e.g., flexion, extension) at similar points in the stroke or stride cycle, with juveniles tending to have slightly higher wingbeat and stride frequencies, and slightly lower duty cycles during the wingstroke (Tables B and C in S1 File). However, the magnitudes of these movements differ across ages, with juveniles showing slightly higher levels of inter-individual variation (Table D in S1 File) but collectively more extreme and exaggerated movements. In the forelimbs, compared to adults, developing birds employ a greater stroke amplitude (#1 in Fig 3 and Figure C in S1 File; S3 Video), hold their wings more extended (#2) and at a higher angle of attack (#3a) during the downstroke, and recover from the downstroke with an exaggerated “scooping” (#4) and “tucking” (#5) motion during the upstroke. For all birds, long axis rotation of the manus occurs roughly in opposition to long axis rotation of the antebrachium (#3b), with juveniles having a greater range of long axis rotation at the wrist than adults (Box A in S1 File). In the hind limbs, juveniles take longer, more lunge-like strides (#1 in Fig 4 and Figure D in S1 File; S4 Video), have a less splayed posture at mid-stance (#2), position their feet more lateral to the body midline (#3), and seem to transition through a series of gaits before becoming adult-like (“lunging” → “speed-skater” → “tight-rope walker”; #4). Developing and adult birds flap-running on similar inclines thus display a number of differences in both fore- and hind limb kinematics (see Table E in S1 File for significant differences).

### H2: older juveniles and adults on narrow versus wide substrates

Substrate width does not appear to affect forelimb kinematics during WAIR. Older birds may keep their wings more elevated on wider substrates (p≤0.31), but this does not affect forelimb kinematics in any predictable way (Figure E in S1 File, Table F in S1 File), and does not prevent birds from depressing their wings more when necessary (see H3).

### H3: adults on shallow (60–65°) versus steep (70–80°) inclines

Nearly all observed differences between juveniles and adults (H1) are diminished or eliminated when adults flap-run up steeper slopes (70–80°). In the forelimbs (Figure F in S1 File), adults flap-running up 70–80° inclines increase their stroke amplitude to juvenile-like levels, by depressing and retracting their humeri more than when flap-running up shallower inclines (#1 in Fig 3 and Figure C in S1 File). During the downstroke, adults keep their wrists more extended (#2), and supinate their antebrachia more–presumably to adopt a higher angle of attack (#3). During the upstroke, adults “scoop” (#4) and “tuck” (#5) their wings more, by supinating their humeri and antebrachia more, abducting their antebrachia more, flexing their elbows more, and adducting their manus more. Similarly, in the hind limbs (Figure F in S1 File), adults on steep inclines take long, more lunging and more juvenile-like steps compared to adults on shallow inclines (#1 in Fig 4 and Figure D in S1 File), by protracting and retracting their femora more, flexing their knees more, and flexing and extending their ankles more. Adults also abduct their femora less (#2), and position their feet more laterally (#3) by adjusting long axis rotation of the femur (more medial rotation) and tibiotarsus (more lateral rotation). Thus, nearly all differences observed between juveniles and adults flap-running on relatively shallow inclines (#’s 1–5 and 1–3 in Figs 3 and 4) are reduced, or disappear, when juveniles flap-running on shallow inclines (60–65°) are compared to adults flap-running on steeper (70–80°), more challenging inclines (see Table E in S1 File for p-values).

## Discussion

Despite having extremely underdeveloped musculoskeletal apparatuses and incipient wings [10], our results indicate that developing birds acquire an “avian” flight stroke early in ontogeny (part (i) below), initially using their wings and legs cooperatively and, as they acquire flight capacity, meeting ontogenetic increases in aerodynamic output with greater skeletal channelization (part (ii)). These findings have important implications for understanding the development of flight in extant birds, and reconstructing the origin of flight in extinct theropods (part (iii)). Integrating skeletal kinematics with previous work on locomotor ontogeny, we posit that aerodynamically active, flapping forelimbs may be adaptations or exaptations for enhancing leg performance, and that wing-enhanced leg performance could have been an important impetus for increases in pectoral muscle mass and improvements in aerodynamic output.

### (i) Developing birds with rudimentary anatomies acquire an avian flight stroke early in ontogeny

When flap-running up the same incline, juveniles and adults have similar patterns but different magnitudes of joint movement: juveniles have more exaggerated or more extreme movements than adults (Figs 3 and 4, Figures C and D in S1 File). In the forelimbs, juveniles have greater stroke amplitudes (#1 in Fig 3), and extend their wings more during the downstroke (#2) but scoop (#4) and tuck (#5) their wings more during the upstroke. Whereas adults pronate their antebrachia ~10° in downstroke and supinate their manus, juveniles supinate their antebrachia up to 30° –achieving higher mid-wing angles of attack [61]–and pronate their manus (#3) (Box A in S1 File). In the hind limbs, juveniles take long, more lunging strides (# 1 in Fig 4), adduct their femora more (#2) in the middle of the stride cycle, and position their feet more laterally (#3). Collectively, these differences suggest that flap-running up 60–65° inclines presents a greater challenge to juveniles than to adults.

Birds meet aerial challenges in a variety of ways. When forced to take off vertically or presented with greater physical demands during flight, birds respond by (i) increasing their stroke amplitude and increasing the disc area swept by the wings during downstroke [70], (ii) flapping at a higher wingbeat frequency [71], (iii) orienting their wings at a high angle of attack [72], presumably to increase resultant aerodynamic forces [18], and/or (iv) tucking in their wings during the upstroke, potentially to reduce drag and inertia [73] when stroke amplitudes and wingbeat frequencies are high. Compared to adults, juveniles (i) sweep out a proportionally larger area during the downstroke by having a greater stroke amplitude (#1 in Fig 3) and extending their wrists to the fullest extent (#2), (iii) use a higher angle of attack (#3) [61], and (iv) tuck their wings in more during the upstroke (#’s 4,5). Forelimb kinematics therefore suggest that at 60–65°, juveniles are performing closer to maximal levels than adults.

Juveniles also appear to be recruiting their hind limbs more closely to maximal levels. Animals challenged to ascend an incline often position themselves closer to the substrate, adopting a more crouched posture (cats (*Felis domesticus*) [74], squirrel monkeys (*Saimiri*) [75]) or pitching the trunk towards the substrate (woodpeckers (Piciformes) [76]). This helps prevent falling backward when traversing steep obstacles, by keeping the center of gravity anterior to the hind limbs and reducing pitching moments. During ascents, cats and squirrel monkeys also increase limb extension at the end of stance, presumably to provide additional propulsion, just as galliforms and ratites extend their limbs more at the end of stance at higher running speeds [77]. Finally, irrespective of incline, animals can improve balance in challenging situations by positioning their feet more laterally to increase the size of the support polygon (area between feet in which center of mass must lie to achieve static stability) [78,79]. Compared to adults, juvenile chukars not only flap-run with their bellies closer to the substrate (lower hip height, more steeply pitched trunk (Table C in S1 File)), but also extend their limbs more at the end of stance (#1 in Fig 4), and position their feet more laterally (#3).

Taken together, these patterns in fore- and hind limb kinematics thus suggest that juveniles and adults perform similar behaviors at different levels of effort. This is consistent with previous work showing that 65° inclines should elicit greater effort in younger birds: 65° is the maximum angle of ascent via WAIR for 7 day old chukars, whereas adult chukars can flap-run up inverted surfaces [53]. Further, when adult chukars flap-run up steeper and more challenging angles (or fly upward at the same angle [60]), they “revert” to more juvenile-like fore- and hind limb kinematics (H3) (Figures C, D, and F in S1 File; Table E in S1 File). Ontogenetic trends or differences in wing and leg kinematics are thus largely reduced or eliminated when juveniles and adults are compared at similar levels of effort, indicating that juvenile birds with rudimentary, “dinosaur-like” anatomies are capable of surprisingly bird-like kinematics (i.e., juveniles and adults have similar kinematics for similar levels of effort). In spite of having small muscles, incipient wings, and relatively gracile and unchannelized skeletons, developing chukars can produce all elements of the avian flight stroke. How is this possible?

### (ii) Developing birds with rudimentary flight apparatuses achieve bird-like wingstrokes initially by using their wings and legs cooperatively, and, as they acquire flight capacity, counteracting ontogenetic increases in aerodynamic output with greater skeletal channelization

When wings and legs are viewed in isolation (wings for aerial locomotion, legs for terrestrial), it is difficult to imagine how animals lacking flight aptations could produce useful aerodynamic forces, other than to slow aerial descents [54]. However, “transitional” flapping behaviors that involve cooperative use of wings and legs (e.g., WAIR, steaming, jumping into brief flapping flights; https://www.youtube.com/watch?v=3USAC-Ky25s) require less muscle power [80] and less aerodynamic force [18,56] than level flight. Transitional behaviors therefore allow flight-incapable juveniles to seamlessly transition to flight-capable adults, supplementing their underdeveloped wings and flight muscles with their legs until the flight apparatus can fully support body weight. By reducing force and power requirements on the forelimbs, hind limb contributions to weight support and propulsion may also allow developing birds to perform complex, bird-like flapping kinematics early in ontogeny, in the absence of flight aptations.

For example, in the axial skeleton, developing chukars lack the fully fused vertebrae and rigid trunk of adults (Fig 1B and 1C) but engage their wings and legs simultaneously, analogous to a quadruped, and do not need to transmit as much wing-generated force to the rest of the body. Smaller aerodynamic forces (<10% body weight in 6–8 day old chicks versus ~60% in adults [18,56]; Fig 2)–and presumably smaller muscle forces (wing muscles ~8% body mass in 7–8 day old chicks versus ~27% in adults [20])–translate to less torque about wing joints. This may prevent the relatively unchannelized forelimb joints of juvenile birds (Fig 1G) from abducting or pronating out of planar alignment during the downstroke. As juveniles increase aerodynamic output and acquire full flight capacity, their skeletal joints become more channelized (less flexible, due to increased ossification of interlocking skeletal components (e.g., v-shaped ulnare) [81] and/or greater concentration of tendons/ligaments crossing joints [AMH, personal observation]) and presumably help resist increases in torque about joints. Thus, while adults probably prevent excessive abduction and long axis rotation mainly by having channelized wing joints, juveniles may achieve the same effect by recruiting their legs and reducing torque about wing joints. Finally, although the underdeveloped flight apparatus of juvenile birds probably results in a more caudally positioned center of mass (caudal to wings’ center of lift), this would not create a pitching moment during WAIR due to a counteracting moment from the hind limbs (contrast level flight, with no hind limb input). Considering the avian body plan in its entirety (wings + legs, skeleton + muscles + feathers) therefore explains why immature chukars lacking many hallmarks of flight capacity can nevertheless produce all elements of the avian flight stroke: their forelimbs produce and resist small amounts of force, and their hind limbs do the rest.

### (iii) Aerodynamically active, flapping wings may be adaptations or exaptations for enhancing leg performance: implications for the development and evolution of avian flight

Many hypotheses have been proposed to explain how flight evolved in the theropod-avian lineage. Regardless of the initial evolutionary route(s) to flight capacity, our data can provide insight into two important topics relevant to all evolutionary scenarios: the role of the hind limbs, and the functional interplay between wing feathers and the musculoskeletal apparatus.

#### Role of the hind limbs

Juvenile birds engage their developing anatomies in an unexpectedly adult-like fashion. Although the unique and specialized morphological features of adults (Fig 1) may be adaptive endpoints for powerful and/or sustained flight (adults display greater wing performance and endurance [18,53,56,61,82] than juveniles without such features), reduced versions of these features do not preclude juveniles from flap-running [53] and using less power-demanding forms of flight (e.g., brief flights with large leg contributions during takeoff [20]), or from flapping with adult-like kinematics. Given that

- all kinematic elements of the avian flight stroke first appear in juvenile birds recruiting their wings and legs cooperatively and generating small but important amounts of aerodynamic force,
- aerodynamic force production first occurs during flapping behaviors that require large contributions from the legs but only small contributions from the wings (e.g., WAIR followed by flapping aerial descents, jumping takeoffs), with incremental increases in wing performance allowing developing birds to flap-run up steeper slopes, or jump higher, and eventually fly, and that
- the developmental and evolutionary acquisition of flight both involve an obligately-bipedal to flight-capable transition [10], marked by continuously functional legs and increasingly functional wings,

we suggest that both the ontogeny and evolution of wing-based locomotion should be considered from a hind limb perspective: aerodynamically active, flapping wings might be better viewed as adaptations or exaptations for enhancing leg performance. Extant birds demonstrate that legs are a crucial component of wing-based locomotion throughout obligately-bipedal to flight-capable transitions, providing the sole source of locomotion in hatchlings (c.f., some megapodes), facilitating pre-flight flapping behaviors in juveniles (e.g., WAIR, steaming, jumping into brief flight), and initiating takeoff in flight-capable adults [10,19,20,53–55,57,83,84]. Just as developing birds must supplement their wings with their legs until flight capable, extinct theropods might have compensated for initial deficits in wing performance (aerodynamic and power output) with ground reaction forces (cursorial hypotheses, Ontogenetic Transitional Wing hypothesis) and/or aerodynamic forces (arboreal hypotheses) from the legs. Hind limb contributions allow developing birds to smoothly transition from (i) leg-based, to (ii) leg+wing-based, to (iii) wing-based behaviors [57], and may have played a similar role during the evolution of flight.

Here, we have focused on legs in the context of wing-assisted incline running, demonstrating how hind limb support allows immature birds with rudimentary musculoskeletal apparatuses and developing wings to generate useful aerodynamic forces with adult-like wingstrokes. Irrespective of how this behavior fits in with the origin of flight (Box 1), developing birds clearly demonstrate that behaviors like WAIR are widespread (https://www.youtube.com/watch?v=k94EDd8aKng), and crucial to survival in animals with rudimentary, developing flight apparatuses. In short, legs offer a fully functional platform for increasingly functional wings to build upon. Though puzzling when considered from a traditional “wings for aerial, legs for terrestrial locomotion” paradigm, partially functional wings become ecologically relevant when viewed from a hind limb perspective.

**Box 1. Evolutionary relevance of WAIR.**

This manuscript suggests that the evolutionary origin of aerodynamically active, flapping wings might be most plausibly viewed as an adaptation or exaptation for enhancing leg performance. In living clades, small but increasing aerodynamic contributions by feathered forelimbs allow developing birds to seamlessly transition from flight incapable juvenile to flight capable adult, initially using their wings to enhance leg performance (e.g., WAIR, wing-assisted jumps) and later co-opting them for flight. Such cooperative use of wings and legs during behaviors like WAIR essentially acts as a developmental bridge between leg-based and wing-based locomotion, allowing juveniles to progress from (i) leg-based, to (ii) leg+wing-based, to (iii) wing-based locomotor behaviors as they acquire the anatomical specializations of adults. Wing-leg cooperation may have played a similar role during the evolution of flight.

Here, we focused on wings and legs in the context of wing-assisted incline running. However, in light of the many recently discovered fossils with long feathers preserved on their hind limbs, some authors have suggested an aerodynamic role for the hind limbs instead, advocating an arboreal, “four-winged” gliding phase as the evolutionary route to avian flight (Table 1 in [10]) and interpreting WAIR as derived and unrelated to the origin of flight (e.g., [85]). Arboreal scenarios often assume that gliding is easier or more efficient than flapping (e.g., [21]), and/or that long hind limb feathers would have hampered terrestrial locomotion [86–88]. However, several observations argue against such views about the evolutionary relevance of WAIR:

Four wings versus two:

- The “four wings” of *Microraptor* [86] are not representative of all paravians, because other paravians and basal avialans with feathered legs (e.g., *Anchiornis*, *Eosinopteryx*, *Archaeopteryx*, *Sapeornis* [43,47,89–91]) generally have shorter, symmetrical hind limb feathers that do not necessarily cover the entire hind limb and that more resemble the feather “trousers” of extant (two-winged) raptors (Figure G in S1 File).
- The nature of the attachment of these leg feathers to the hind limb bones, their internal construction, and their potential ability to form an aerodynamically competent airfoil have never been substantiated [92]. What leg postures would have been necessary for leg feathers to contribute aerodynamically, and would they have been anatomically possible given constraints at the hip and relatively hinge-like ankles? To date, no one has established the range of possible leg postures in extinct paravians, or the function of leg feathers during flight among living birds, in order to to answer these questions.
- If hind limb feathers are aerodynamically advantageous (either for weight support or steering), why were they reduced in avialans [47,90] that still lack the flight-related skeletal features of extant adult birds (e.g., keel), and why do extant birds with extensively feathered legs generally tuck their legs out of the way once airborne [93,94]? Legs and leg feathers create drag, which is often detrimental to flight performance and must be considered.

Gliding versus flapping:

- There is no empirical basis for reconstructing paravians with feathered hind limbs as gliders rather than flappers, even if the only locomotor function of the wings is to slow and control aerial descents. Why do feathered hind limbs preclude flapping? Extensively feathered legs are common among many flying (flapping) birds (Figure G in S1 File).
- Gliding is not necessarily “easier” than flapping, and no one has ever shown that gliding is an evolutionary prerequisite for flapping (in fact, gliders and flappers are nowhere near each other on phylogenetic trees [95]). Flapping aerial descents and wing-assisted incline running can involve very small amounts of aerodynamic force production and power output [18,56,80], and are regularly used by juvenile birds with extremely rudimentary flight apparatuses (protowings [10,17,53] + tiny keel and small flight muscles–mass of pectoral girdle and limbs ~9% in 8 day old chukars [20], ~12% reconstructed in *Archaeopteryx* [36]). Also, flapping precedes gliding during development. All data available to date suggest that flight-incapable juvenile birds (ranging from galliforms [61] to passerines [96]) slow aerial descents by flapping rather than gliding. Even once flight capacity is acquired in species that are specialized for soaring, juveniles rely more heavily on flapping than adults do [97].
- We should not expect new phenotypes and behaviors to be selected first for efficiency, but rather for efficacy. Hovering (hummingbirds) and wing-assisted incline running (chukars, all observed species) are not “efficient” behaviors, either in terms of lift-to-drag ratios [18] or Strouhal numbers ([98]; flapping at extremely low advance ratios is undefined in this sense). Yet both behaviors are highly effective for the animals that use them. WAIR can mean the difference between life and death to a juvenile bird that cannot yet fly, and predator avoidance is an extremely important selective pressure. We should expect new structures and behaviors to evolve first because they are efficacious, and only later to be selected for efficiency.
- Even if some extinct theropods could glide, it cannot be assumed that all did. Extant birds show high interspecific variation in locomotor behavior, and there is no reason to expect that avian predecessors were restricted to one style of locomotion. Extant birds also demonstrate that individuals use many different habitats (foraging on ground, roosting in trees, etc.) and engage in many different locomotor activities. No living birds only glide (to the best of our knowledge), some birds only flap (e.g., hummingbirds, except for diving display flights), and most birds both glide and flap (although gliding is often interspersed with flapping, as during intermittent flight (e.g., [99])). Dichotomizing extinct theropods as either exclusive arboreal gliders or exclusive terrestrial flappers is simply not realistic [100].

Hind limb feathers versus terrestrial locomotion:

- Hind limb feathers do not actually inhibit terrestrial activities any more than they inhibit arboreal locomotion. Extant birds with completely feathered hind limbs (e.g., some pigeons, chickens, raptors) are capable of running and jumping (S5 Video). There is no evidence that extensive hind limb feathers inhibit terrestrial locomotion.

Phylogenetic generality of WAIR:

- Wing-assisted incline running is no more derived than powered flight: given the phylogenetic distribution of this behavior (https://www.youtube.com/watch?v=k94EDd8aKng), it is conservative to hypothesize that the common ancestor of birds that flap their wings to fly was also able to flap-run. WAIR is actually less derived than gliding: birds specialized for soaring (e.g., albatrosses, raptors, vultures) are more derived than many birds that flap-run (e.g., galliforms and tinamous).

In short, many assumptions about WAIR are incorrect and there is no reason to dismiss WAIR as irrelevant to the origin of flight. Basal paravians and basal avialans show substantial variation in feather [10,43,86,89,91,101] and pedal [102] morphology, and although they were not as diverse as extant birds [103,104], everything known about locomotion in living animals suggests that basal paravians and avialans would have used their wings and legs for a variety of non-locomotor and locomotor purposes–including leg-based behaviors like WAIR–as they experimented with volancy. We may never be able to pinpoint a “single behavior” that led to the origin of flight, and it is probably not realistic to assume that only one behavior was important. What has become clear, however, is that legs are an extremely important component of wing-based locomotion in birds, and likely played an important role in the beginnings of flapping and powered flight [10,19,20,90,105]. A central problem in the origin of flight is the origin of the flight stroke, which requires a precise kinematic movement with an aerodynamically competent wing [106], and no theory of the origin of flight can be satisfactory without explaining the evolution of this stroke.

#### Wing feathers versus the musculoskeletal apparatus

Origin of flight hypotheses are often expressed in a “ground-up” / flapping versus “trees-down” / gliding framework. Irrespective of one’s stance on this topic, flapping locomotion clearly evolved at some point in the theropod-avian lineage. Flapping probably did not originate in animals with fully bird-like flight apparatuses, because the evolution of some avian features simply cannot be explained in the absence of flapping locomotion. Whereas feathered forelimbs serve both locomotor and non-locomotor (e.g., thermoregulation, display [37–43], brooding [44,45], stability [46]) functions that likely contributed to the wing (feather) evolution, hypertrophied pectoral muscles and associated skeletal attachment sites are only associated with powered, flapping flight (as far as we know), and thus likely evolved in response to selection for greater aerodynamic output via wing flapping. In other words, multiple selective pressures likely contributed to the evolution of forelimb feathers, and, once aerodynamic functions came into play, selection for greater aerodynamic output via wing flapping would have resulted in hypertrophied pectoral musculature and associated skeletal attachments. Consistent with this sequence, bird-like wings appear before bird-like skeletons in the fossil record (and in developing chukars; summarized in Figures 1 and 2 of [10]). Evolutionary hypotheses must therefore either address the functional interplay between rudimentary musculoskeletal apparatuses and aerodynamically active, flapping forelimbs, or provide an alternative, viable explanation for the evolution of hypertrophied musculoskeletal apparatuses in the absence of flapping locomotion.

Here, we have explored how immature birds flap their developing wings to improve leg performance. By reducing force and power requirements on the forelimbs, hind limb contributions to weight support and propulsion seem to allow young birds to perform complex, bird-like flapping kinematics in the absence of many flight aptations. Hind limb input becomes less necessary as more adult-like anatomies are acquired. This helps clarify avian ontogeny and a crucial phase of flight evolution relevant to all origin of flight scenarios, by elucidating the functional interplay between rudimentary musculoskeletal apparatuses (all juveniles) and flapping feathered forelimbs (protowings (7–8 days old) or wings (18–20 days old)), and by demonstrating how selection for wing-enhanced leg performance could have been an important impetus for increases in muscle mass and improvements in aerodynamic output.

## Conclusions

Developing birds demonstrate that many morphological features long viewed as adaptations or exaptations for flight are not necessary for flapping behaviors such as wing-assisted incline running or jumping into brief flight. Immature chukars with rudimentary musculoskeletal apparatuses and developing wings achieve an avian flight stroke early in ontogeny, initially by using their wings and legs cooperatively and reducing force and power requirements on the forelimbs. Leg contributions become less crucial as juveniles acquire the specialized anatomies of adults and become more capable of producing aerodynamic force and resisting associated torques about wing joints. Thus, while the unique morphological features of adult birds may be adaptive endpoints for powerful and/or sustained flight, rudimentary versions of these features do not preclude immature birds from flap-running and using less power-demanding forms of flight, or from flapping with adult-like kinematics. In conjunction with previous studies, our findings therefore show that wing-leg cooperation is a behavioral and functional bridge between leg- and wing-based modes of locomotion. Feathered forelimbs serve many functions, but from a locomotor perspective, work with developing birds suggests that the initial function of developing wings is to enhance leg performance, and that aerodynamically active, flapping wings may be adaptations or exaptations for enhancing leg performance.

## Supporting Information

S1 File All supporting tables, figures, and boxes. (PDF)

S1 Video X-ray footage of chukar partridges engaged in wing-assisted incline running. 7–8 day old, 11–12 day old, and 18 day old birds flap-running on 60–65° inclines, in lateral view; adult bird flap-running on 65–80° inclines (65° data from [60]).
(MOV)

S2 Video Rotoscoped x-ray footage of chukar partridges engaged in wing-assisted incline running. Rotoscoped forelimbs and hind limbs of 7–8 day old and 18 day old birds flap-running on 60–65° inclines, in lateral and dorsal view.
(MOV)

S3 Video Ontogeny of forelimb kinematics. Mean downstroke and upstroke kinematics for 7–8 day old (red), 11–12 day old (orange), 18 day old (green), and adult (purple) birds engaged in wing-assisted incline running on 60–65° inclines.
(MOV)

S4 Video Ontogeny of hind limb kinematics. Mean stance and swing kinematics for 7–8 day old (red), 11–12 day old (orange), 18 day old (green), and adult (purple) birds engaged in wing-assisted incline running on 60–65° inclines.
(MOV)

S5 Video Wing-assisted incline running in a chick with long leg feathers. (MOV)
