# Supplementary material for: Flapping before Flight: High Resolution, Three-Dimensional Skeletal Kinematics of Wings and Legs during Avian Development
Source: PLoS One. 2016 Apr 21;11(4):e0153446. doi: 10.1371/journal.pone.0153446 (PMC4872793; doi:10.1371/journal.pone.0153446)
Supplement: S1 File — (PDF) [file pone.0153446.s001.pdf]

Table of Contents

Table A. Experimental setup	2
Table B. Forelimb kinematics	3
Table C. Hindlimb kinematics	4
Table D. Standard deviations for each joint rotation, averaged across stroke or stride cycle	5
Table E. Ontogenetic trends and differences between juveniles and adults (H1, H3)	6
Table F. Effect of ramp width (H2)	7
Figure A. Skeletal models	8
Figure B. Rotoscoping versus Marker-Based XROMM: Validation	9-10
Figure C. Forelimb kinematics (H1, H3)	11
Supporting Information for Figure C and Table E	12-20
Figure D. Hindlimb kinematics (H1, H3)	21
Supporting Information for Figure D and Table E	22-32
Figure E. Effect of ramp width (H2)	33
Figure F. Adults flap-running on shallow versus steeply angled ramps	34
Figure G. Long leg feathers in extant birds	35
Box A. Washout	36
References	37

Table A. Experimental setup

Age class (dph)	Morphology				Locomotor capacity	Experimental treatment, technique, sample size
	Body mass (g)	Wings and Feathers	Muscle mass (% body mass)	Skeleton		
7-8	28-30	Small protowings with large gaps between distally unfurled juvenile feathers	F: 8%<br>H: 11%	Partially ossified skeleton with highly flexible joints and extremely small keel	CFD<br>65° WAIR	1, SR, n=3
11-12	38-41	Protowings with small gaps between juvenile feathers	x		CFD<br>75° WAIR	1, SR, n=3
18-23	68-89	Adult-like wings with juvenile feathers	F: 18%<br>H: 13%		CFD<br>85-90° WAIR<br>Brief flight	18: 1, SR, n=3<br>23: 1+2, SR, n=2<br>(multiple trials)
Adult (≥100)	400<br>-<br>600	Full sized wings	F: 27%<br>H: 18%	Fully ossified skeleton with channelized (restricted) joints and large keel	CFD<br>>100° WAIR<br>Flight	1, SR, n=2<br>(multiple trials)<br>3, SR+MbX, n=1<br>(multiple trials)

dph: days post hatching; F: forelimbs; H: hindlimbs; SR: Scientific Rotoscoping [1]; MbX: Marker-based XROMM [2]; n: sample size (refers to number of individuals per treatment, not necessarily number of wingbeats or stride cycles - see Materials and Methods)

Treatment 1 (60-65°, wide ramp): ramp angled at 60-65° (depending on ability of bird), ramp width = 23-28 cm (≥ length between left and right manus when wings fully extended at mid-downstroke)
Treatment 2 (60-65°, narrow ramp): same as 1, except ramp width = 11 cm (allowing wingtips to extend over edge of ramp)
Treatment 3 (70-80°, wide ramp): same as 1, except ramp angled at 70-80°

Body mass data from [3], feather morphology from [4], muscle mass from [5], locomotor capacity from [3, 6, 7], adult Treatment 1 data from [8]. Note that in [8] the ramp angle was set to 70°; however, this study used coarse-grit sandpaper, which enhances traction and is therefore equivalent to 65° WAIR on medium-grit sandpaper (i.e., birds began flap-running consistently at 70° rather than at 65°).

Table B. Forelimb kinematics

**Age** (dph)	**Wingbeat frequency** (cycles/s)		**Duty factor** (% of cycle spent in downstroke)	
**7-8**	14.4	0.3	49.5	2.9
**11-12**	13.7	2.3	47.7	2.0
**18**	14.8	0.8	47.7	1.4
**Adult (shallow WAIR)**	13.5	0.5	51.3	2.8
**Adult (steep WAIR)**	13.8	1.4	47.8	3.6

dph: days post hatching; for wingbeat frequency and duty factor, left sub-column is the value of interest, right sub-column is one standard deviation. Note that standard deviations are either for an age class (juvenile birds with one measurement per bird) or for individuals (adult birds with repeated measurements).

Table C. Hindlimb kinematics

**Age** (dph)	**Stride frequency** (cycles/s)		**Duty factor** (% of cycle spent in stance)		**Hip height during stance** (% max)		**Pitch angle during stance** (0): parallel to ramp (-): into ramp	
**7-8**	5.0	1.6	66.5	5.2	46.9	9.9	-30.4	2.7
**11-12**	5.2	0.1	60.3	5.1	41.6	7.2	-32.5	6.5
**18**	5.4	1.8	56.4	2.8	65.8	13.5	-29.9	5.1
**Adult (shallow)**	3.4	0.0	63.8	2.4	62.8	11.9	-10.4	0.4
**Adult (steep)**	4.7	0.6	49.4	0.1	48.0	2.3	-27.0	12.0

dph: days post hatching; for stride frequency, duty factor, hip height, and pitch angle, left sub-column is the value of interest, right sub-column is one standard deviation. Note that standard deviations are either for an age class (juvenile and adult (shallow WAIR) birds with one measurement per bird) or for individuals (adult birds (steep WAIR) with repeated measurements).

Table D. Standard deviations (in degrees) for each joint rotation (x, y, z), averaged across stroke or stride cycle

Joint		Age				
		7-8	11-12	18	Adult, shallow	Adult, steep
Shoulder	x	14.5	16.2	10.6	5.6	10.6
	y	8.8	9.8	8.4	6.0	6.8
	z	12.0	11.0	9.8	7.4	8.6
Elbow	x	10.4	7.4	8.0	6.3	8.3
	y	5.7	5.6	6.1	6.0	4.6
	z	11.0	12.3	10.1	6.7	5.8
Wrist	x	8.7	9.2	14.0	14.3	15.3
	y	10.4	9.5	9.5	8.8	6.9
	z	12.1	14.6	13.8	15.5	8.0
**Forelimb Average**		**10.4**	**10.6**	**10.0**	**8.5**	**8.3**
Hip	x	8.5	6.5	7.0	2.5	4.9
	y	5.0	4.3	4.8	2.1	2.3
	z	11.7	8.5	14.0	5.6	7.5
Knee	x	10.7	13.2	10.4	6.6	7.2
	y	6.2	6.3	6.2	3.1	4.1
	z	11.6	7.4	24.4	8.5	11.4
Ankle	z	13.7	8.6	27.8	6.5	8.8
**Hindlimb Average**		**9.6**	**7.8**	**13.5**	**5.0**	**6.6**

x: long axis rotation, y: protraction-retraction (shoulder) or abduction-adduction (elbow, wrist, hip, knee), z: elevation-depression (shoulder) or flexion-extension (elbow, wrist, all leg joints)

Adults tend to be slightly less variable than juveniles, and hindlimb movements tend to be slightly less variable than forelimb movements.

Table E. Ontogenetic trends and differences between juveniles and adults (H1, H3)

				Ontogenetic trends?				Differences between adults (A) and juveniles (J)?			Differences between adults at shallow (A) vs steep (As) angles?			
				Spearman's rho		Mixed Effects		A-J	p		As-J	As-A	p	
Joint	#	rotation		r_s	p	slope	p		Welch's t	Tukey	Is |As-J| < |A-J|?	Does As move towards J?	Welch's t	Tukey
**Shoulder**	1	z	Min	0.42	0.16	4.71	0.31	18.12	**0.01**	**0.02**	-7.58	-25.70	**0.00**	**0.00**
	1	y	Min	0.81	**0.00**	7.95	**0.02**	15.97	**0.00**	**0.00**	5.58	-10.40	**0.01**	**0.00**
	4	x	Max	-0.28	0.36	-2.13	0.42	-12.26	**0.00**	**0.00**	-0.40	11.85	**0.00**	**0.00**
**Elbow**	5	z	Min	0.71	**0.01**	7.67	0.08	25.94	**0.00**	**0.00**	9.82	-16.12	**0.02**	**0.00**
	4	y	Avg	-0.54	0.06	-1.93	0.11	-6.25	**0.03**	**0.00**	11.73	17.99	**0.00**	**0.00**
	3a, 4	x	Avg	-0.66	**0.01**	-7.69	**0.02**	-29.72	**0.00**	**0.00**	-21.09	8.63	**0.02**	**0.02**
	4		Range	-0.77	**0.00**	-7.46	**0.03**	-19.82	**0.01**	**0.00**	-9.58	10.24	0.12	**0.04**
**Wrist**	2	z	Max	-0.49	0.09	-8.21	0.26	-49.15	**0.01**	**0.00**	-16.16	32.99	**0.01**	**0.00**
	6		Min	-0.66	**0.01**	-12.97	0.06	-9.26	0.34	0.41	-35.66	-26.40	0.96	1.00
	5	y	Min	0.82	**0.00**	8.22	**0.04**	22.26	**0.00**	**0.00**	0.15	-22.11	**0.02**	**0.00**
	3b	x	Range	-0.48	0.09	-4.82	0.17	-16.93	**0.04**	**0.00**	37.36	54.29	**0.04**	**0.00**
**Hip**	1a	z	Max	-0.24	0.42	0.92	0.64	-6.69	**0.02**	0.06	-0.89	5.81	0.18	**0.05**
	1b		Min	0.85	**0.00**	4.94	**0.02**	7.13	**0.05**	**0.05**	2.06	-5.06	0.13	**0.05**
	2	y	Avg	0.44	0.18	1.87	0.30	6.71	**0.00**	**0.00**	0.96	-5.75	**0.00**	**0.00**
	2		Min	0.60	**0.03**	1.91	0.28	12.64	**0.00**	**0.00**	3.66	-8.98	**0.00**	**0.00**
	3	x stance	Avg	0.09	0.79	-2.90	0.26	-11.03	**0.01**	0.09	-7.51	3.52	0.19	0.13
**Knee**	1a	z	Min	0.28	0.38	1.71	0.70	25.37	**0.00**	**0.00**	16.57	-8.80	**0.05**	**0.00**
	3	x stance	Avg	-0.11	0.74	-1.22	0.82	17.55	**0.01**	**0.03**	9.29	-8.26	0.10	**0.03**
**Ankle**	1b	z	Max	-0.62	**0.02**	-7.81	**0.03**	-7.29	0.08	0.15	3.16	10.45	**0.04**	**0.00**
	1a		Min	0.30	0.38	4.71	0.28	20.69	**0.00**	**0.00**	9.59	-11.10	**0.01**	**0.00**

#: # in Figs 3,4, C, D; rotation: joint rotations shown in Figs C,D (z, top row: elevation-depression (shoulder) or flexion-extension (elbow, wrist, all leg joints); y, middle row: protraction-retraction (shoulder) or abduction-adduction (elbow, wrist, hip, knee); x, bottom row: long axis rotation); Avg (average), Max (maximum), Min (minimum), and Range: kinematic variables tested for statistical significance (non-significant kinematic variables not reported, but full table available upon request). Significant p-values shown in bold (rounded to two decimal places); cells highlighted blue indicate whether patterns are best explained as an ontogenetic trend or as a difference between adults and juveniles (adults versus all juveniles); cells highlighted green indicate that adults flap-running at steep angles are kinematically more similar to juveniles than adults flap-running at shallow angles are, for the kinematic variable in question.

Ontogenetic trends tested using Spearman's rho (does not account for bird ID) and Mixed Effects Models (account for bird ID); ontogenetic differences (adults versus all juveniles), and differences between adults flap-running at shallow versus steep angles, tested using Welch's t-tests (does not account for bird ID) and Tukey Contrasts on Mixed Effects Models (accounts for bird ID) - for full details, see Materials and Methods. Note that Spearman's rho and Welch's t-tests generally give similar results to Mixed Effects Models and Tukey Contrasts, respectively.

Table F. Effect of ramp width (H2)

Dorsoventral distance between sternum and tip of manus (% trunk length; wide ramp - narrow ramp)	p-value	
	Welch's t	Tukey
15.16	0.31	**0.04**

Dorsoventral distance between substrate and tip of manus (% trunk length; wide ramp - narrow ramp)	p-value	
	Welch's t	Tukey
36.05	0.08	**0.00**

Refer to Fig E for discussion

Figure A. Skeletal models

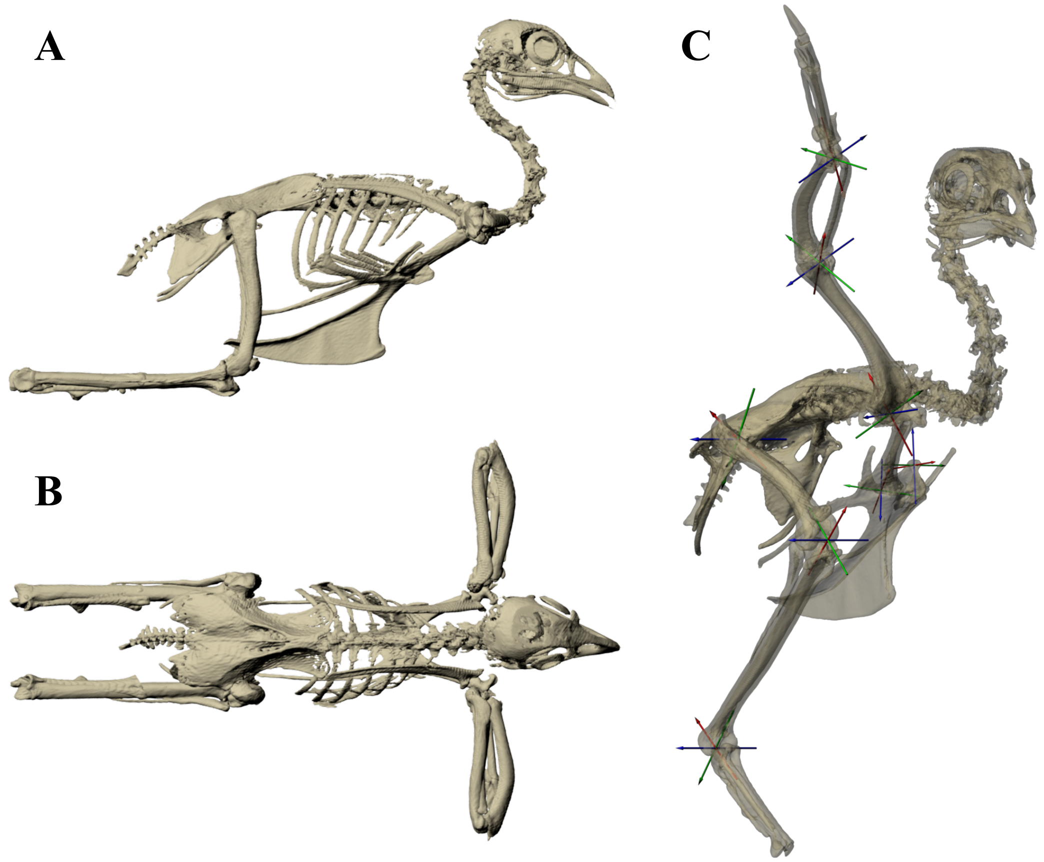

Starting "zero" positions and orientations for each joint in an adult chukar, in lateral (A) and dorsal (B) view. Joint coordinate systems (C) for the pelvis (whole body motion, not shown) and sternal, coracosternal, shoulder, elbow, wrist, hip, knee, and ankle joints defined using inertial axes and anatomical landmarks, as in (8), such that each joint is allowed 6 degrees of freedom (3 translational, 3 rotational) and rotation occurs first around the z-axis (blue), then y-axis (green), then x-axis (red) (xyz rotation order in Maya). Negative rotations listed first:

Joint	Location	z-axis	y-axis	x-axis
Pelvic	center of ventral pelvis	yaw	pitch	roll
Sternal	episternal process	yaw	pitch	roll
Coracosternal	center of sternal facet	adduction-abduction	protraction-retraction	long axis rotation
Shoulder	center of humeral head	depression-elevation	retraction-protraction	pronation-supination
Elbow	btwn dorsal, ventral humeral condyles	flexion-extension	adduction-abduction	pronation-supination
Wrist	center of ulnare	flexion-extension	adduction-abduction	pronation-supination
Hip	center of femoral head	retraction-protraction	adduction-abduction	lateral rotation - medial rotation
Knee	btwn medial, lateral femoral condyles	flexion-extension	adduction-abduction	lateral rotation - medial roation
Ankle	btwn medial, lateral tibial condyles	flexion-extension	adduction-abduction	lateral rotation - medial roation

Starting positions and joint coordinate systems defined identically in juveniles and adults.

Figure B. Rotoscoping versus Marker-Based XROMM: Validation

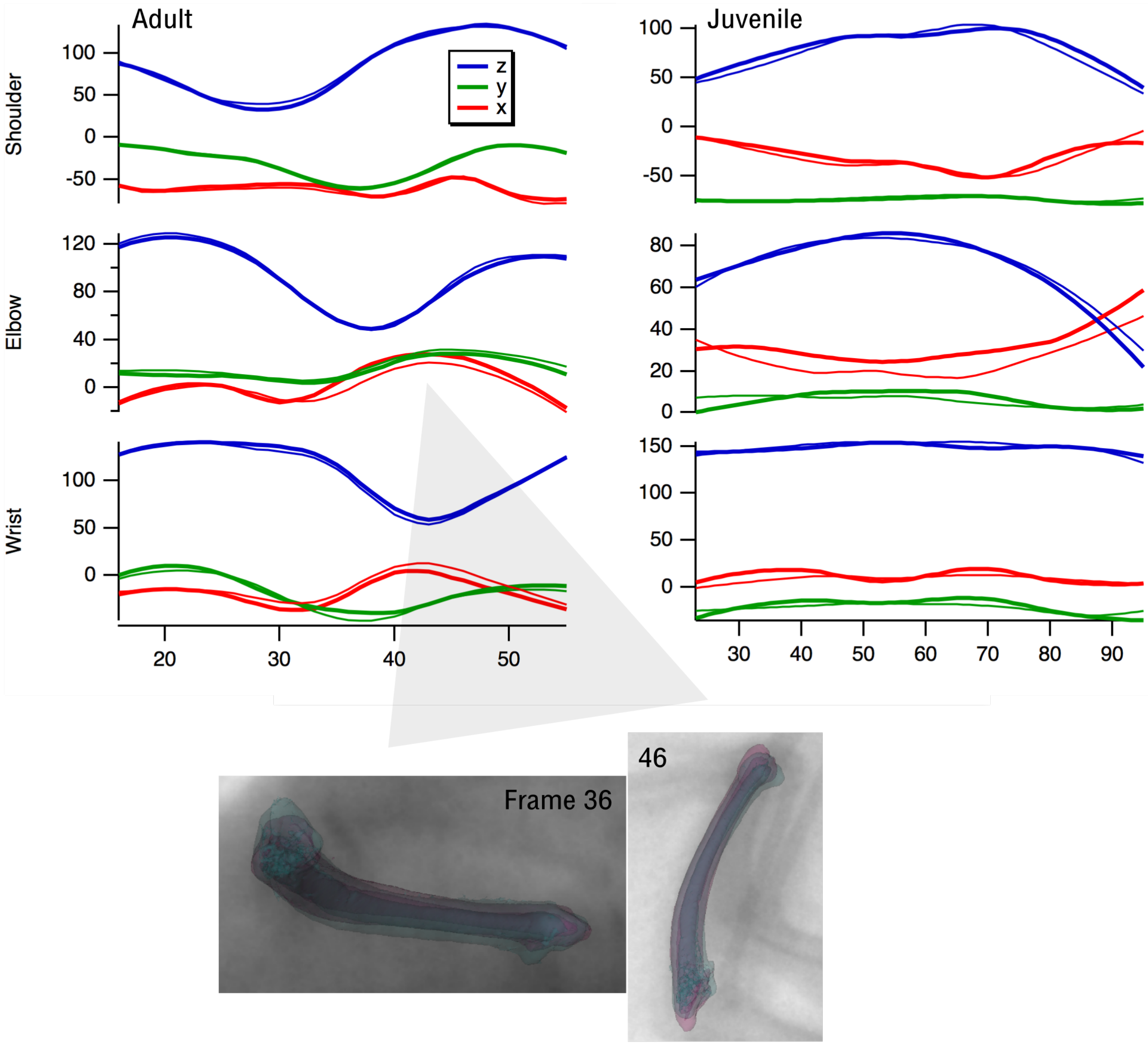

Marker-based XROMM rotations in dark lines, rotoscoped rotations in light lines, with adult forelimbs on left and 9 dph forelimbs on right; adult rotations are for downstroke followed by upstroke, juvenile rotations are for “upstroke” followed by “downstroke” (see Materials and Methods). z: elevation-depression (shoulder) or flexion-extension (elbow, wrist), y: protraction-retraction (shoulder) or abduction-adduction (elbow, wrist), x: long axis rotation.

Overall, markerless and marker-based XROMM give very similar results, with adults (mean difference: 1.0-5.5°, SD: 0.8-3.3°) being slightly more similar than juveniles (mean difference: 1.2-7.1°, SD: 1.1-3.2°) (see table below). No rotations are greatly or consistently offset between markerless and marker-based XROMM, other than long-axis rotation of the antebrachium (markerless consistently < marker-based, for both age classes). This offset may be due differences incurred from aligning the ulna by itself (marker-based) versus aligning the radius

and ulna together, as a single unit (rotoscoping); visually the ulna is aligned very similarly for both techniques (see frames 36 and 46 above; different colors represent different techniques), and for rotoscoping, the offset of the ulna does not offset abduction-adduction or pronation-supination of the downstream manus (as would be expected if we had truly rotoscoped long-axis rotation of the ulna poorly). We therefore conclude our rotoscoping error is small and does not impact the observed ontogenetic trends and differences, although we would recommend implanting even a single marker near joints that might be obscured or difficult to see.

Figure C. Forelimb kinematics (H1, H3)

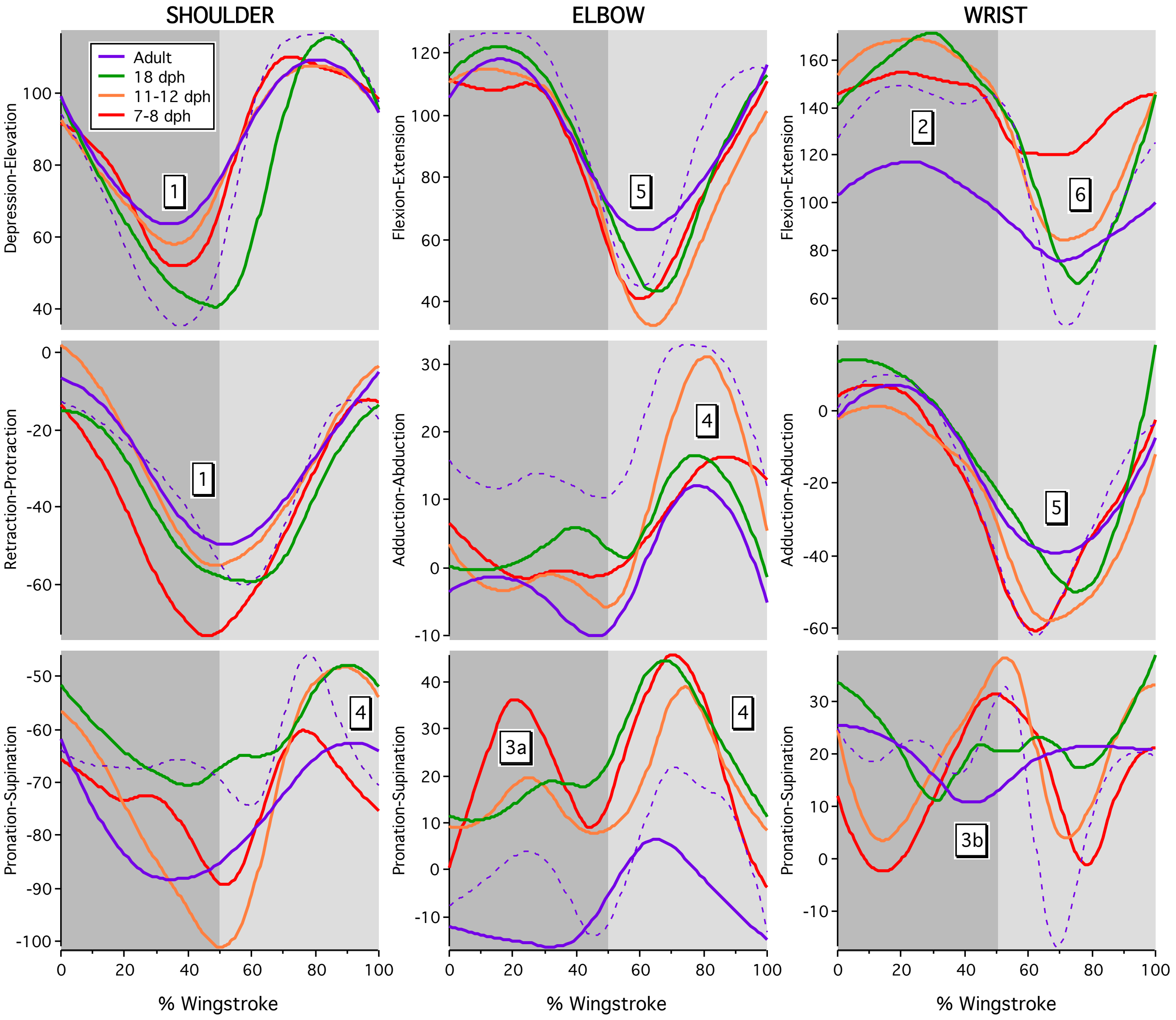

Downstroke shown in dark gray, upstroke in light gray; solid lines indicate WAIR at 60-65° (Treatment 1), dashed lines indicate WAIR at 70-80° (Treatment 3). All lines are age-class averages, with significant differences among age classes indicated by numbers (corresponding to #'s 1-6 in Fig 3).

Supporting Information for Figure C and Table E: pages 12-20

Note that for all of the following plots (pages 12-20), pronation and supination of the carpometacarpus are reversed compared to the plot on page 11

7-8 dph: 65° WAIR

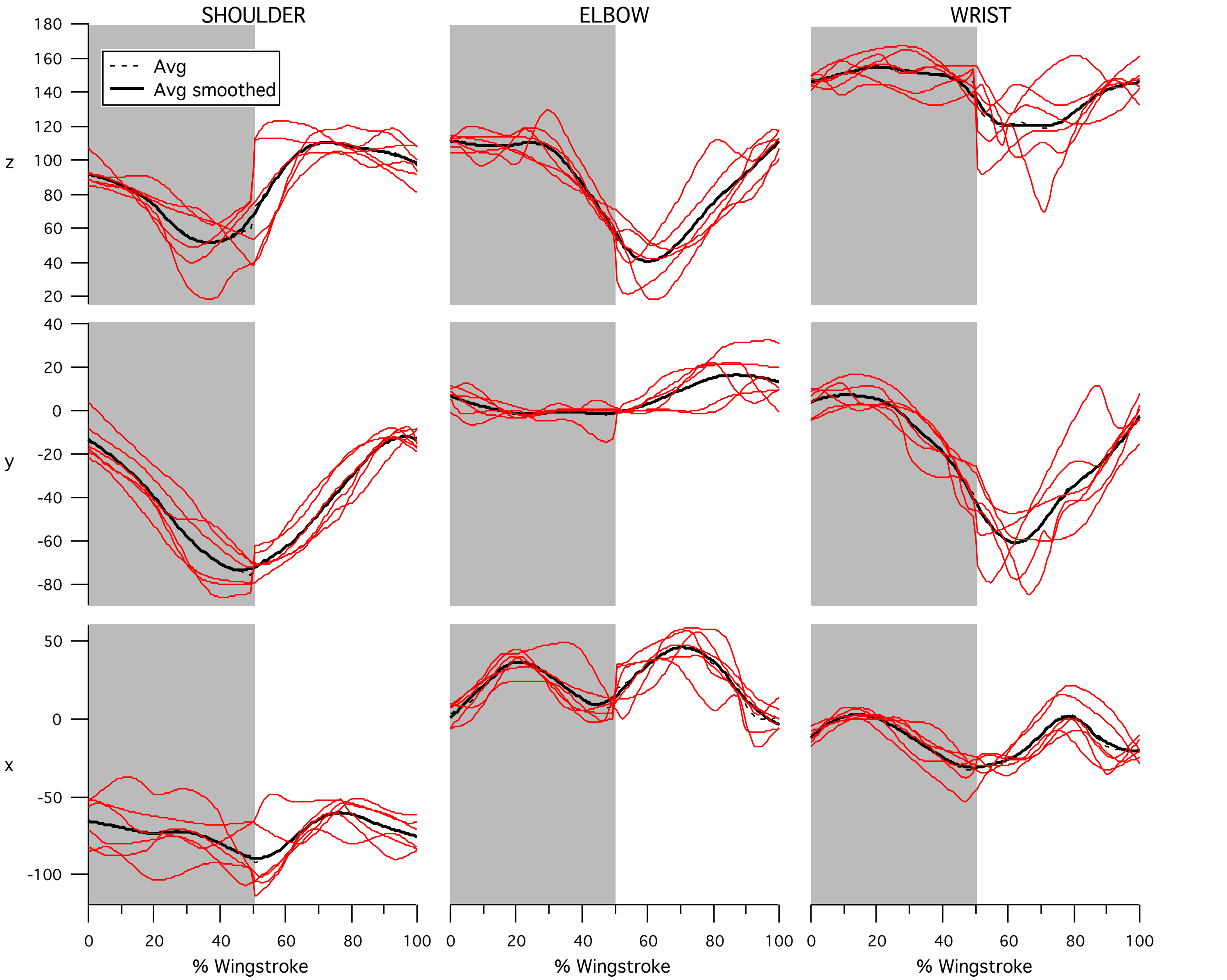

3 birds (mo, mx, pq), one wingstroke each (left + right wing); Loess smooth .3
Note that any abrupt changes at downstroke-upstroke transitions occur because an upstroke then downstroke was rotoscoped for the bird in question, and shown here as a downstroke then upstroke

11-12 dph: 65° WAIR

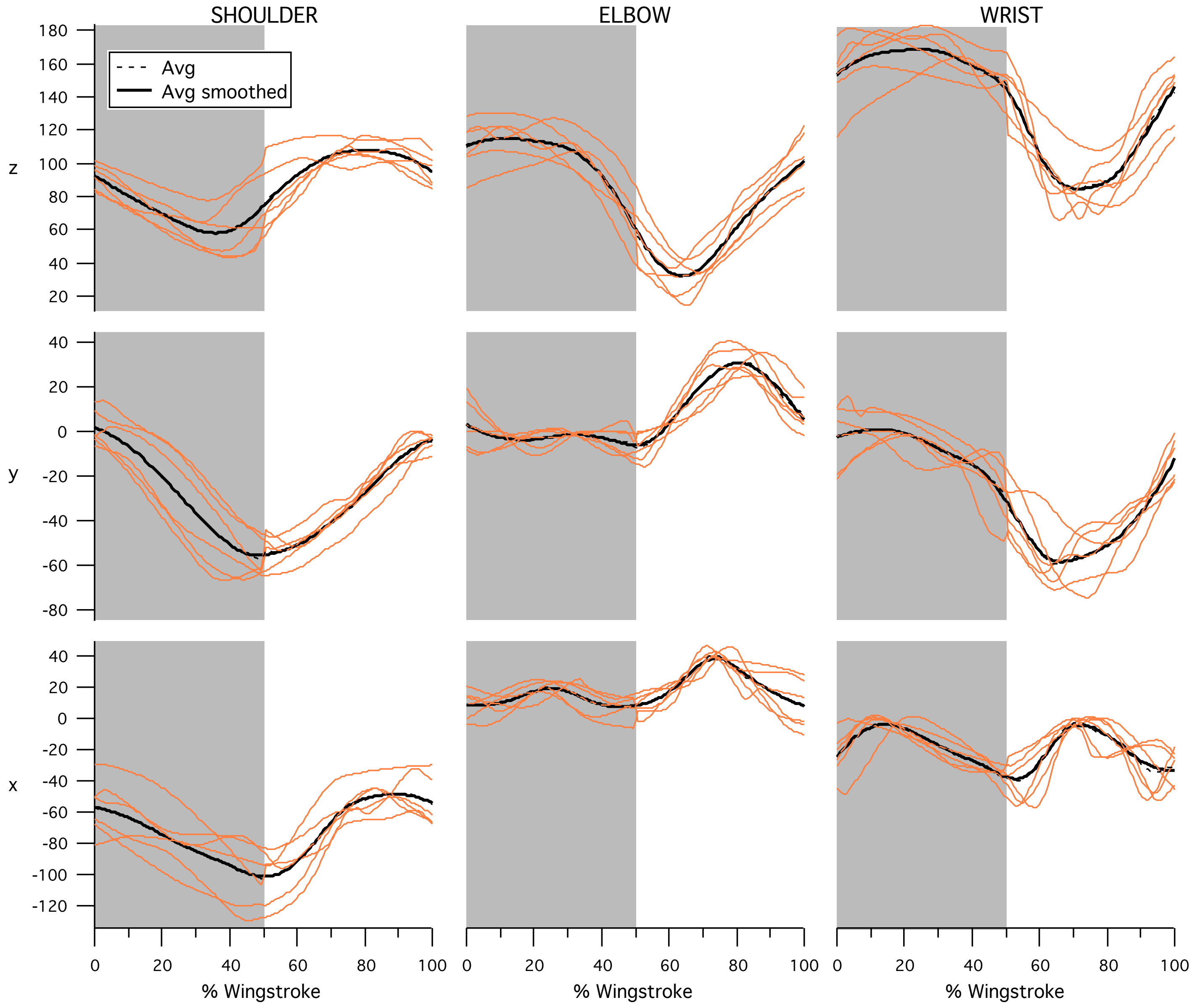

3 birds (mx, pd, pq), one wingstroke each (left + right wing); Loess smooth .3
Note that any abrupt changes at downstroke-upstroke transitions occur because an upstroke then downstroke was rotoscoped for the bird in question, and shown here as a downstroke then upstroke

18 dph: 65° WAIR

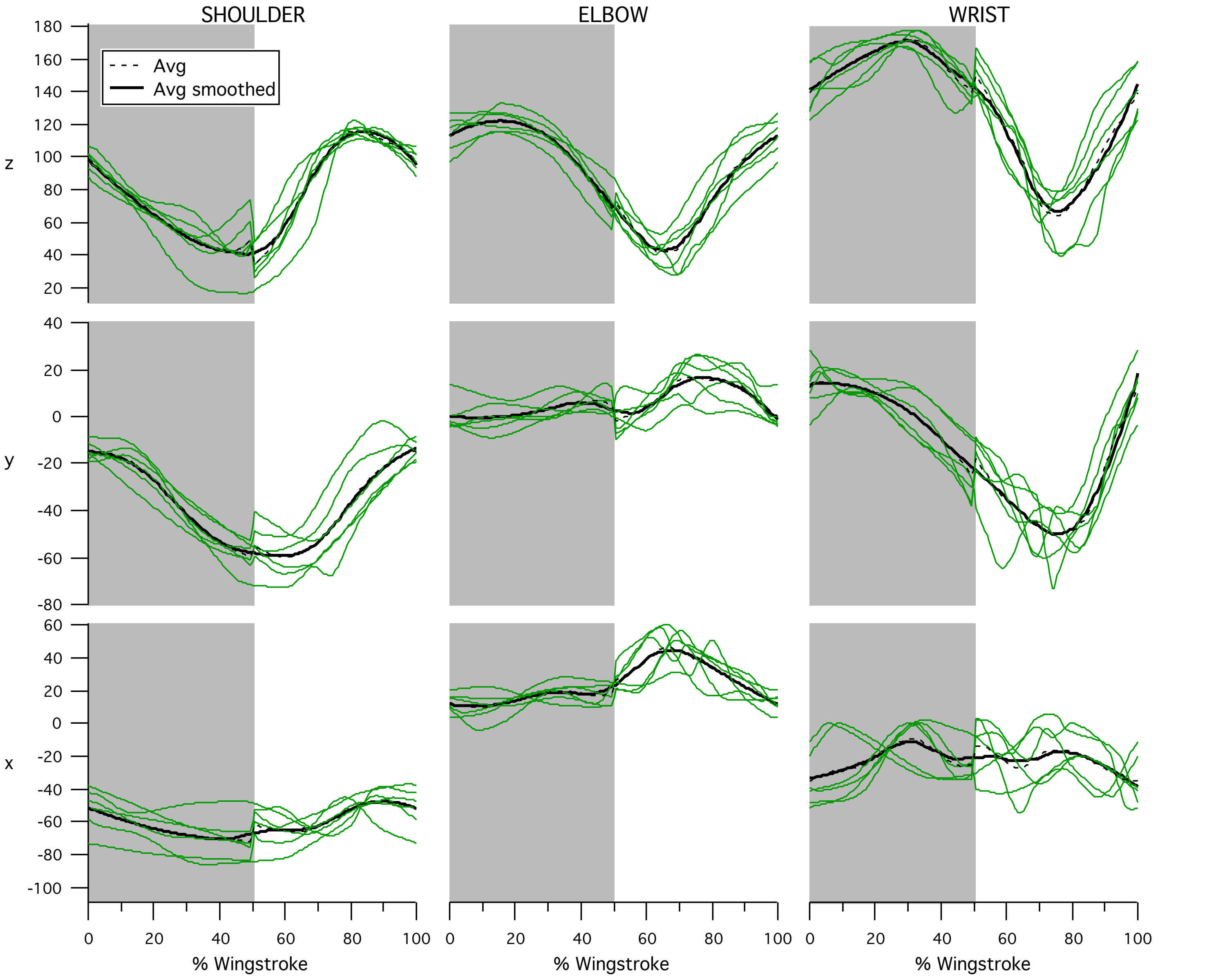

3 birds (bx, rs, rx), one wingstroke each (left + right wing); Loess smooth .4
Note that any abrupt changes at downstroke-upstroke transitions occur because an upstroke then downstroke was rotoscoped for the bird in question, and shown here as a downstroke then upstroke

Adults (>100 dph): 65° WAIR

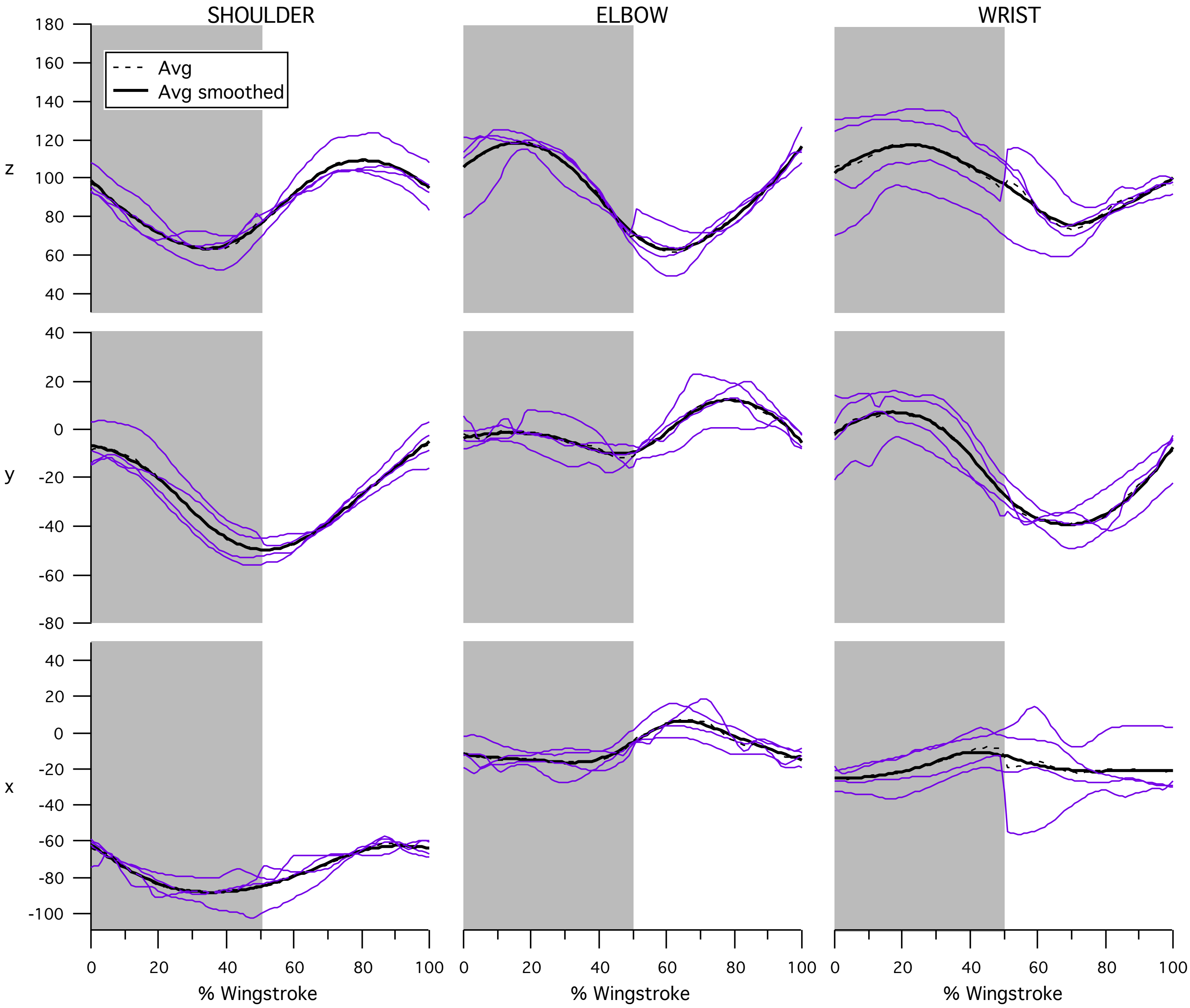

2 birds (Wc3, Wc4), 2 wingbeats each, 2 trials each; Loess smooth .5
Note that any abrupt changes at downstroke-upstroke transitions occur because an upstroke then downstroke was rotoscoped for the bird in question, and shown here as a downstroke then upstroke

Adults (>100 dph): 70-80° WAIR

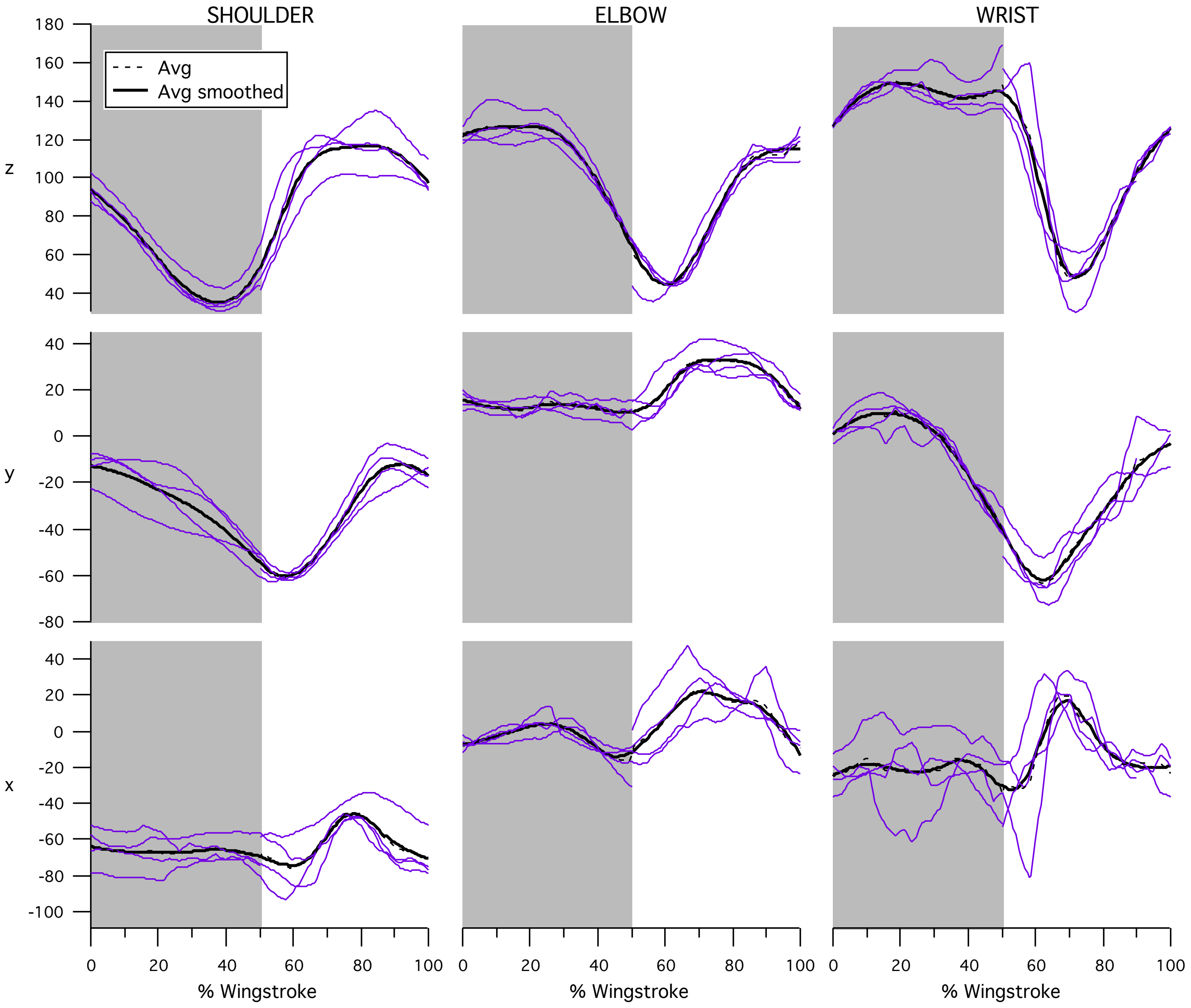

1 bird (cf), 4 wingbeats, 3 trials; Loess smooth .3
Note that any abrupt changes at downstroke-upstroke transitions occur because an upstroke then downstroke was rotoscoped for the bird in question, and shown here as a downstroke then upstroke

ONTOGENETIC TRENDS OR DIFFERENCES? (see Table E)
Points are values for individual birds of a given age: 7-8 dph (rank 1), 11-12 dph (2), 18 dph (3), Adult on shallow ramp (4), Adult on steep ramp (5)

Wing Averages
(Downstroke + Upstroke)

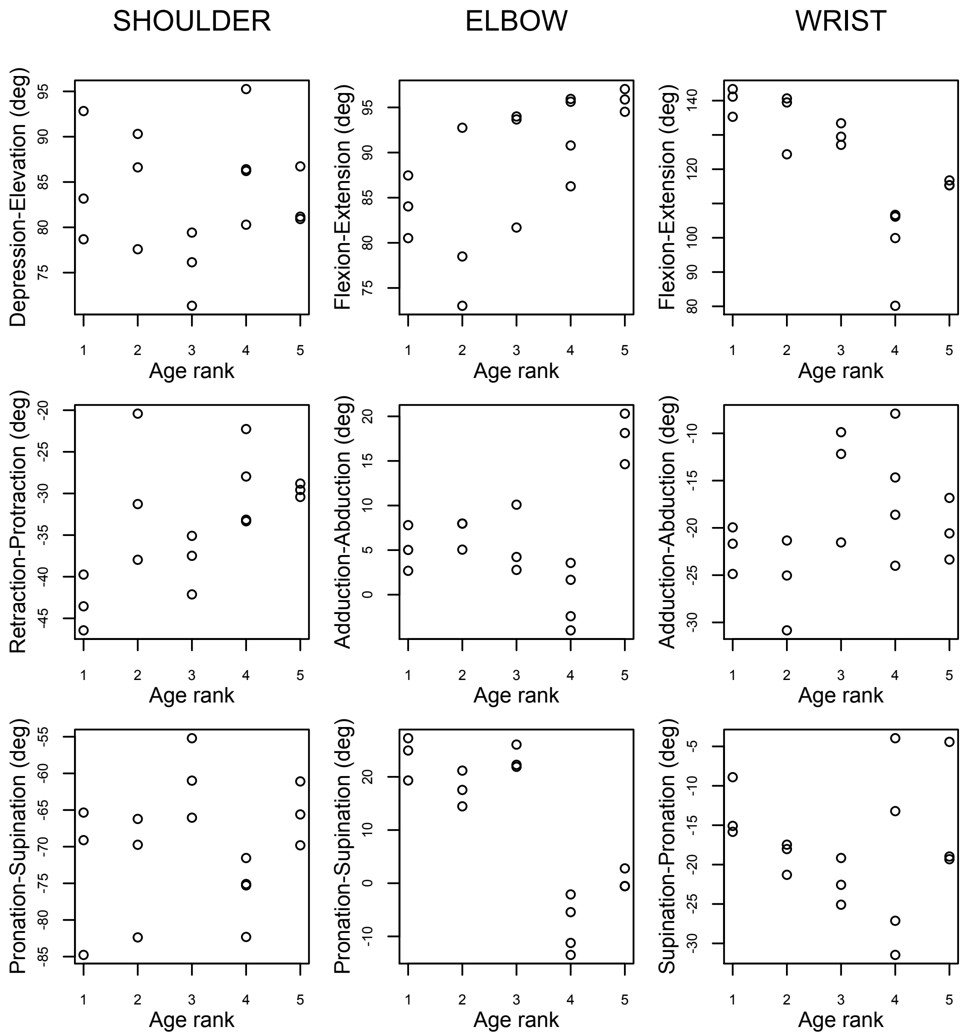

Wing Maxima
(Downstroke + Upstroke)

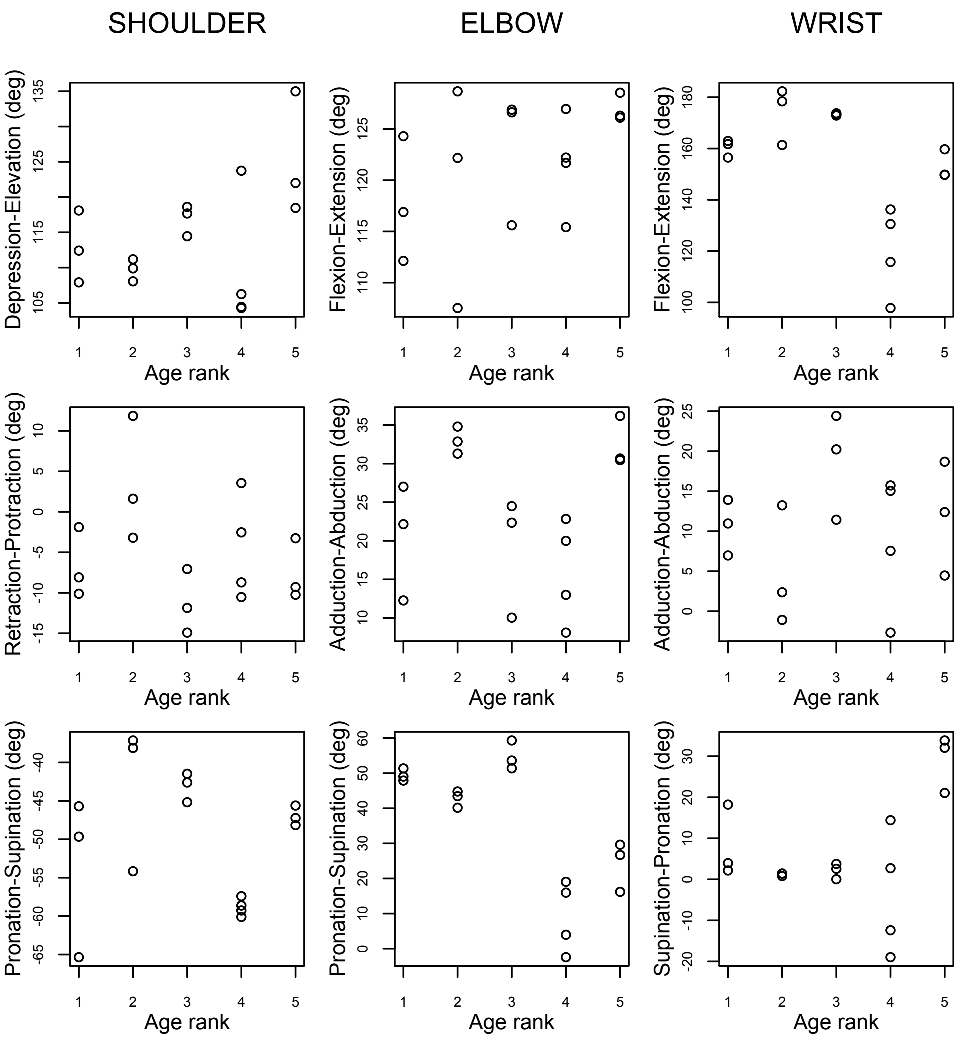

Wing Minima

(Downstroke + Upstroke)

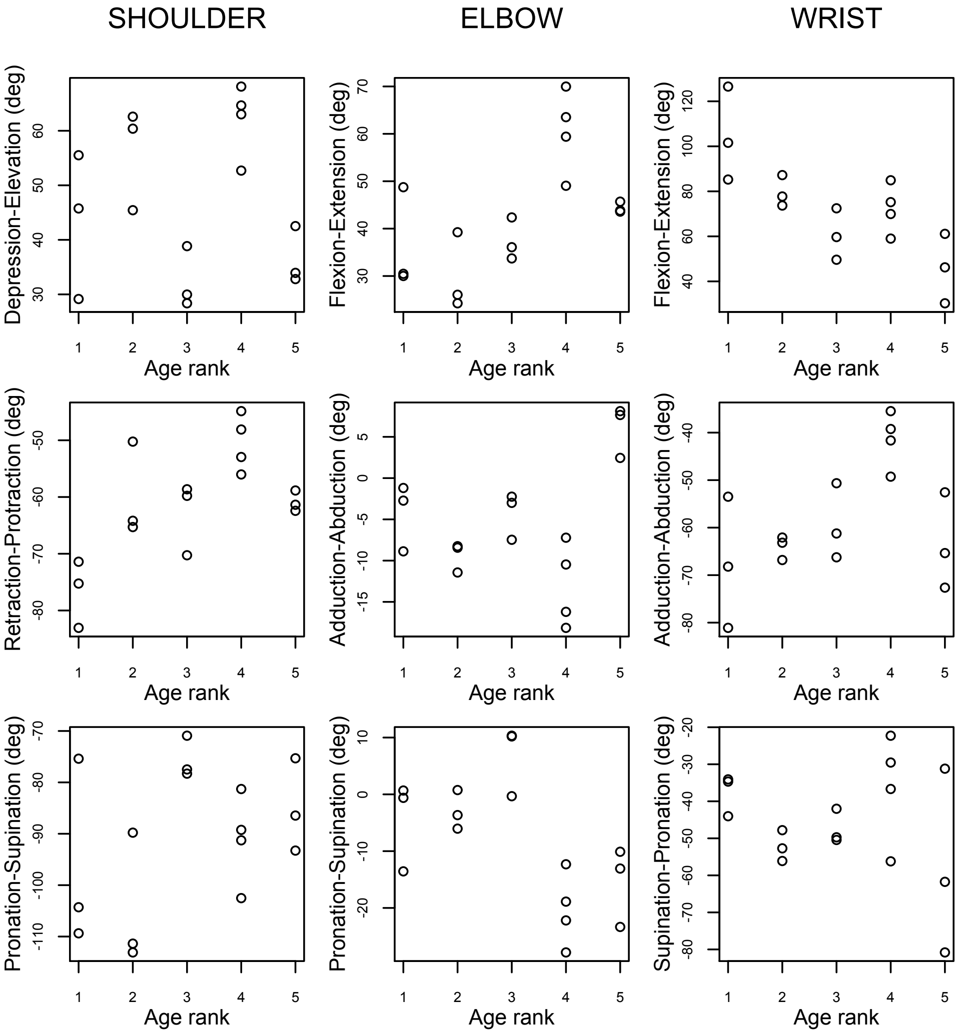

Wing Ranges
(Downstroke + Upstroke)

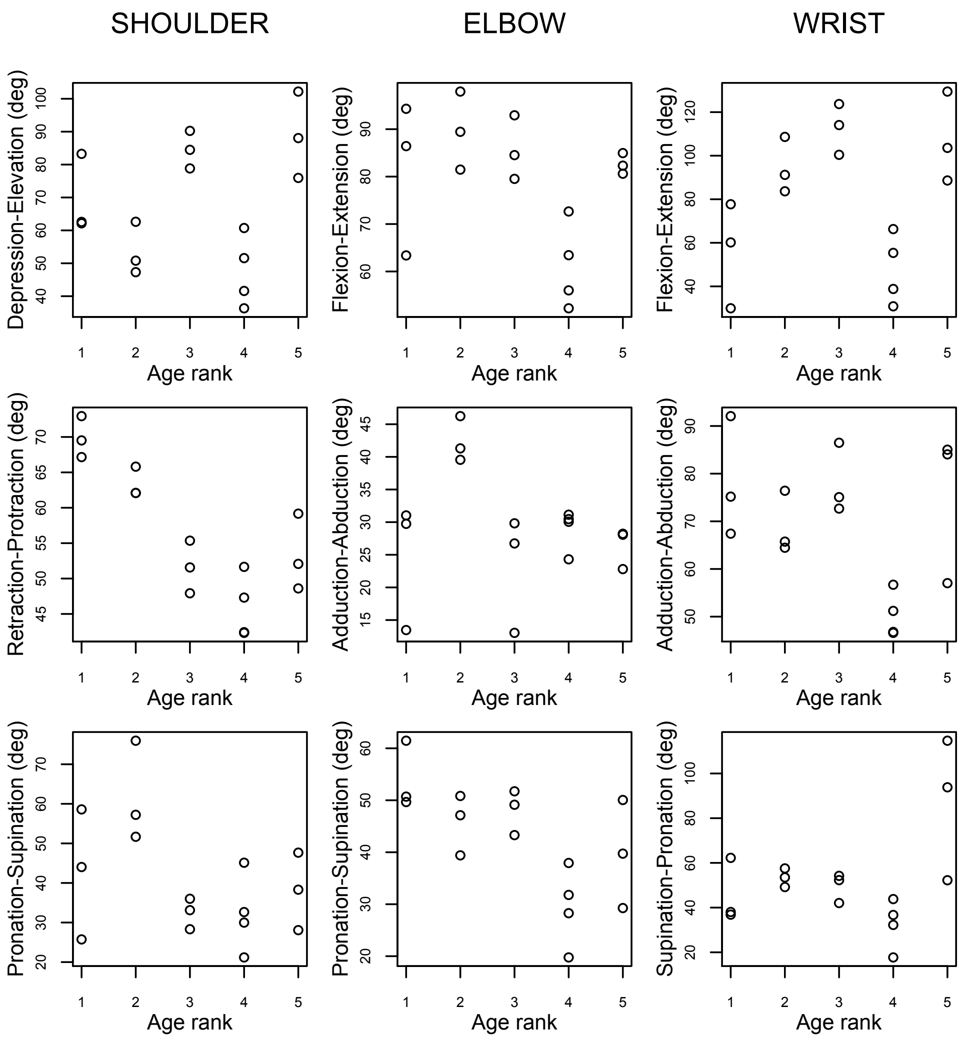

Figure D. Hindlimb kinematics (H1, H3)

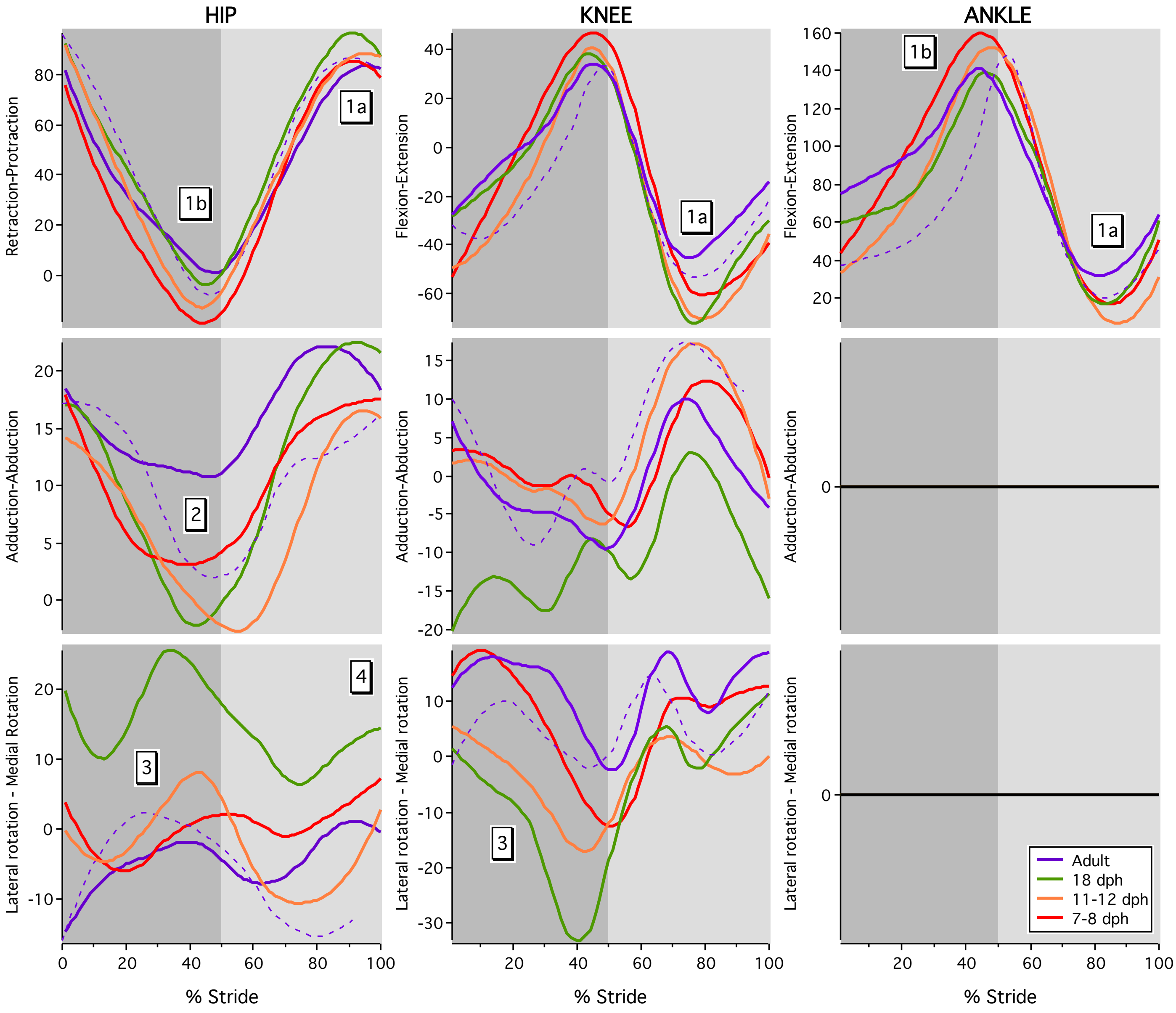

Stance shown in dark gray, swing in light gray; solid lines indicate WAIR at 60-65° (Treatment 1), dashed lines indicate WAIR at 70-80° (Treatment 3). All lines are age-class averages, with significant differences among age classes indicated by numbers (corresponding to #'s 1-4 in Fig 4). Note that any adduction or abduction of the ankle is incorporated into long axis rotation of the tibiotarsus, and that long axis rotation of the ankle was too small to measure; hence, both types of movement are indicated as a flat line (see Materials and Methods for full explanation).

Supporting Information for Figure D and Table E: pages 22-32

Note that for the plots on pages 22-26, ankle flexion and extension are reversed compared to the plot on page 21

7-8 dph: 65° WAIR

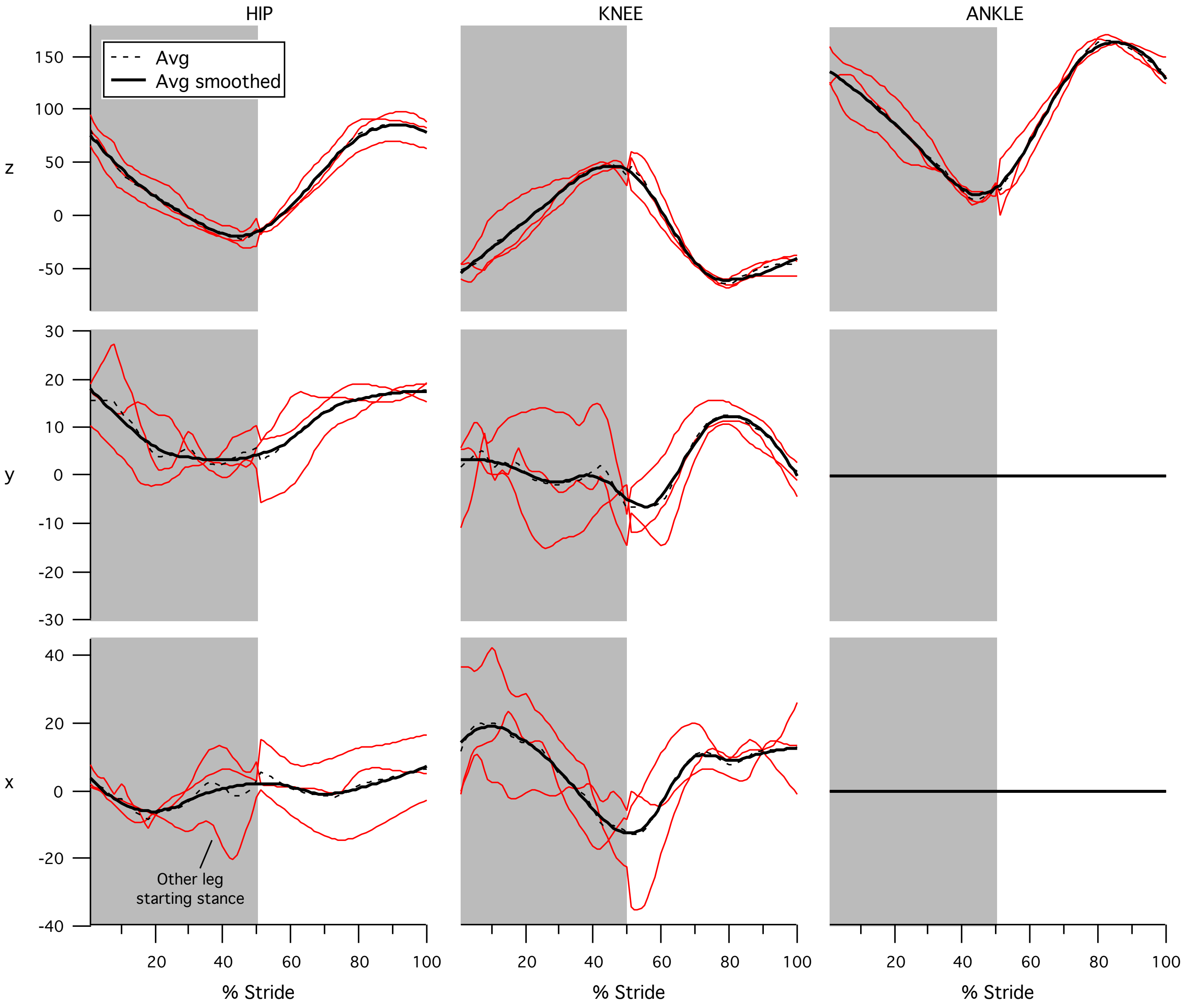

3 birds (mo, mx, pq), one stride each; Loess smooth .3-.5
Note that any abrupt changes at stance-swing transitions occur because stance was rotoscoped for one leg of the bird in question, and swing was rotoscoped for the other leg

11-12 dph: 65° WAIR

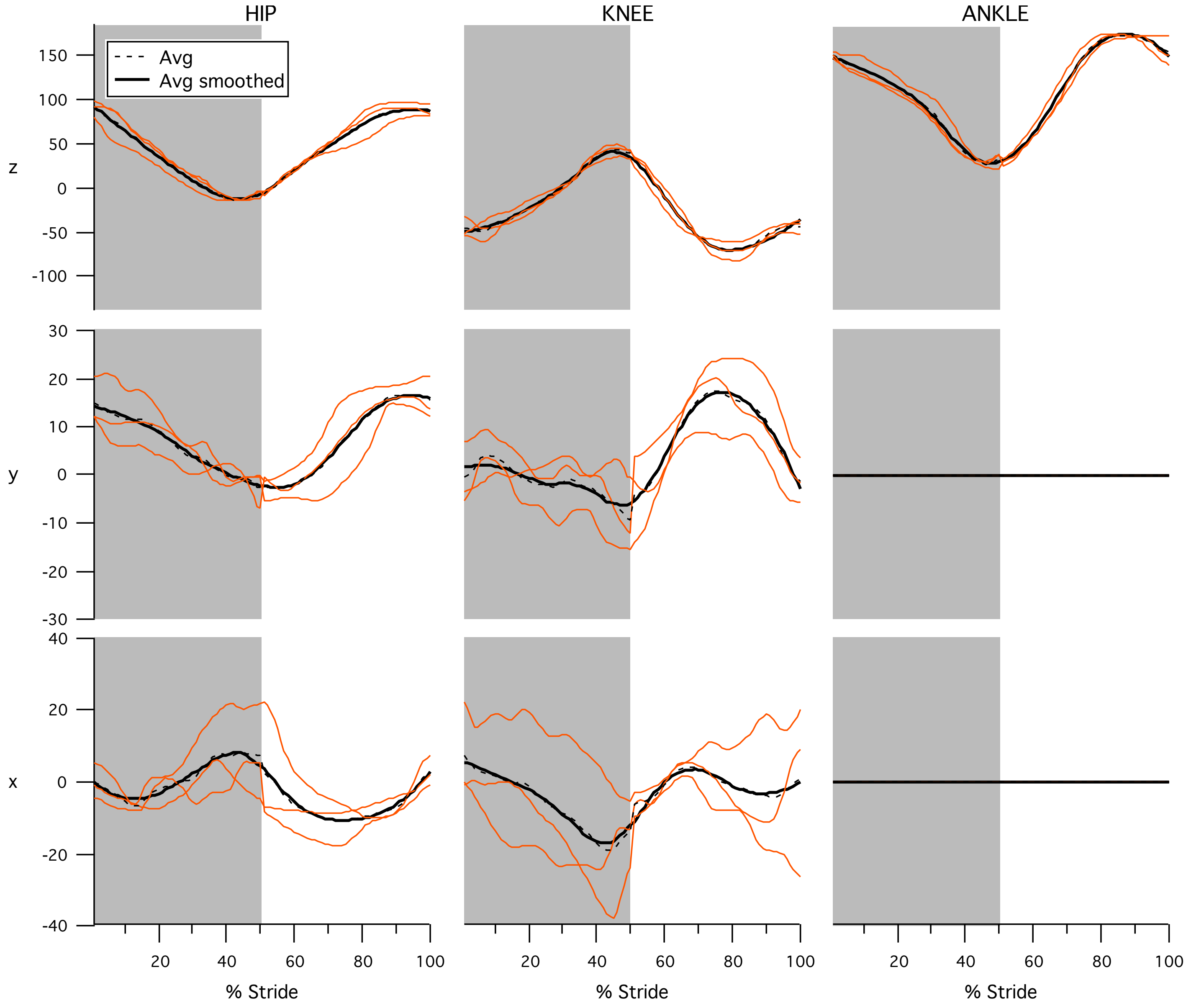

3 birds (mx, pq, pd), one stride each; Loess smooth .4
Note that any abrupt changes at stance-swing transitions occur because stance was rotoscoped for one leg of the bird in question, and swing was rotoscoped for the other leg

18 dph: 65° WAIR

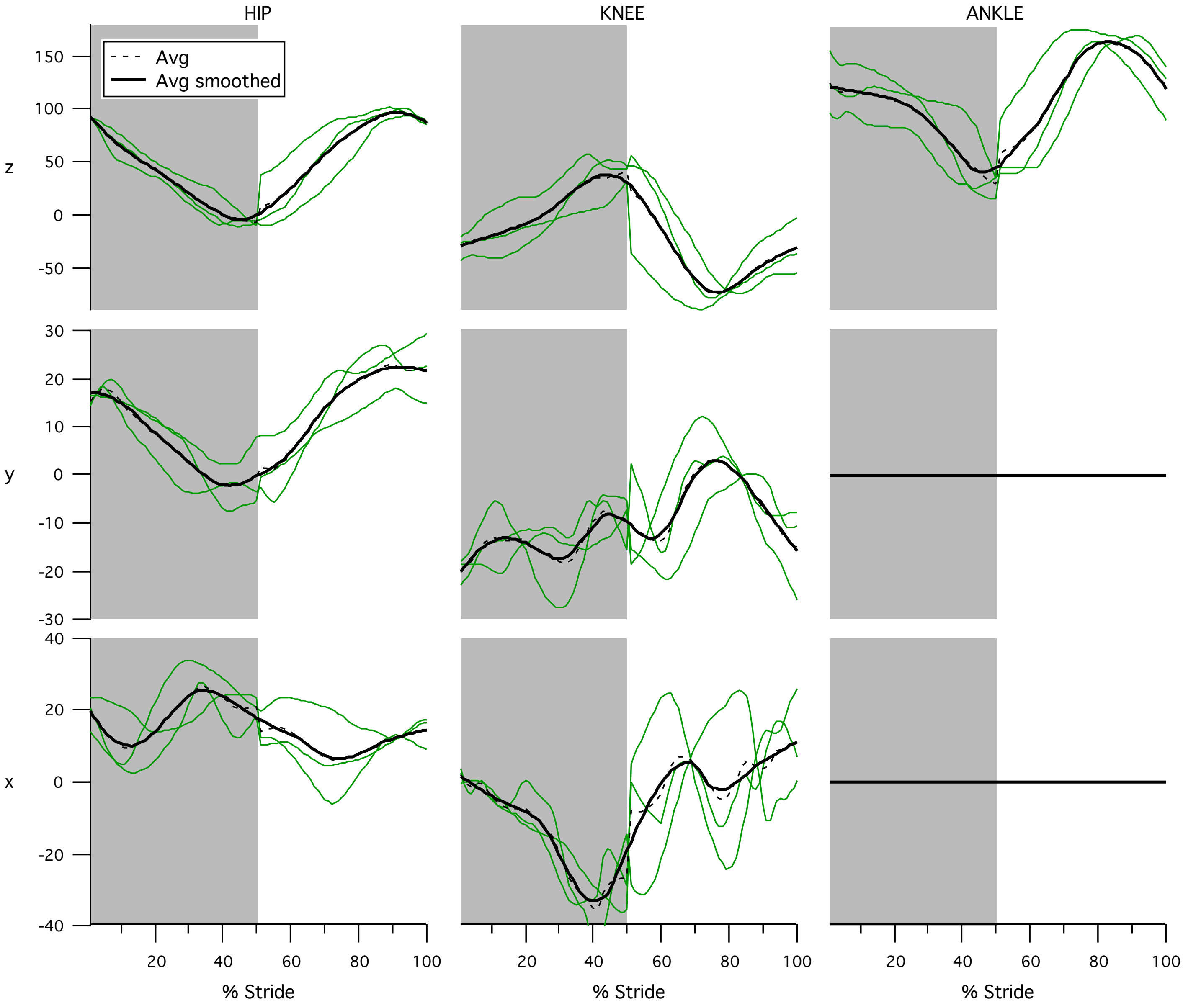

3 birds (bx, ps, rx), one stride cycle each; Loess smooth .3
Note that any abrupt changes at stance-swing transitions occur because stance was rotoscoped for one leg of the bird in question, and swing was rotoscoped for the other leg

Adults (>100 dph): 65° WAIR

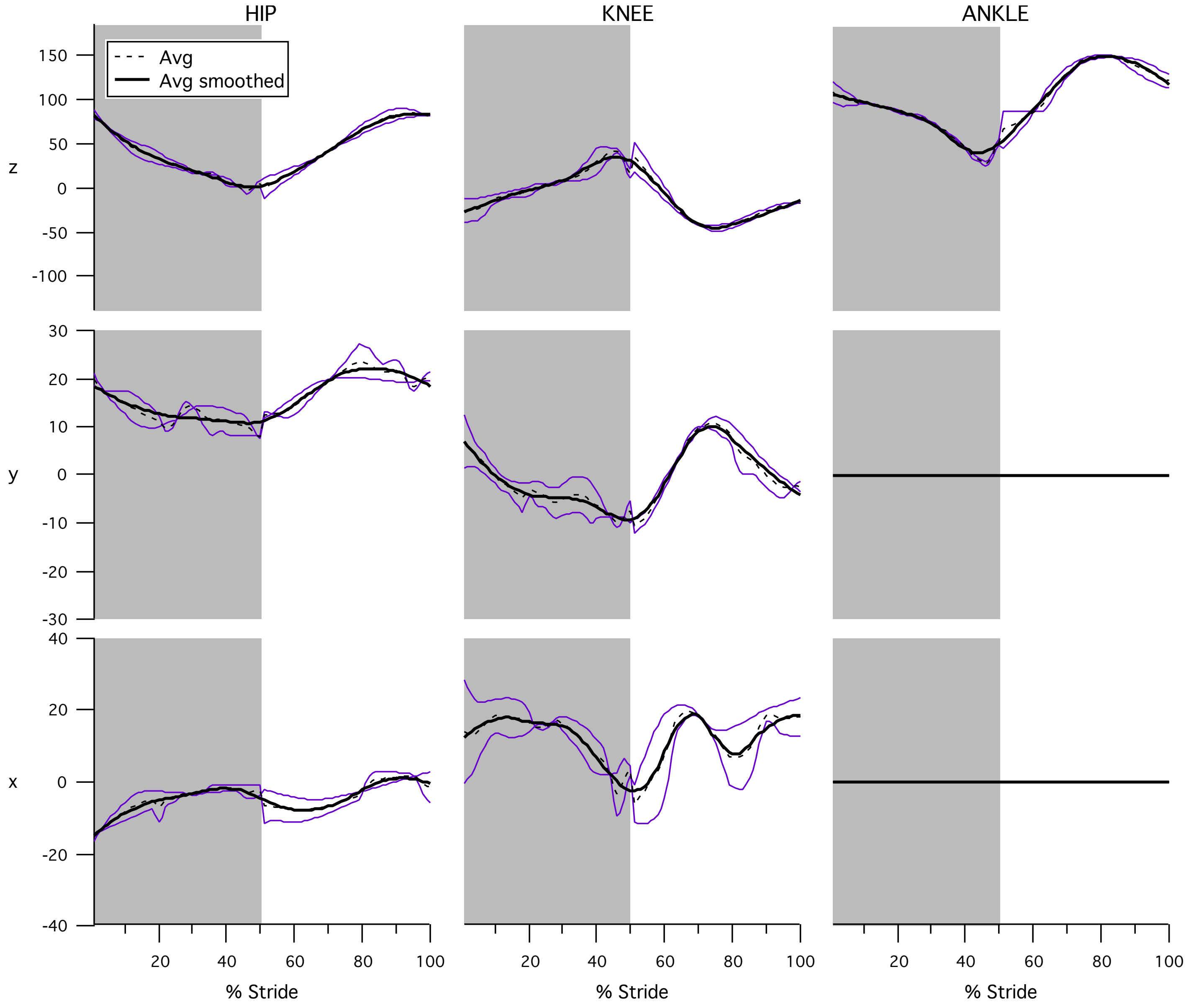

2 birds (c3, c4), one stride cycle each; Loess smooth .3-.6
Note that any abrupt changes at stance-swing transitions occur because stance was rotoscoped for one leg of the bird in question, and swing was rotoscoped for the other leg

Adults (>100 dph): 70-80° WAIR

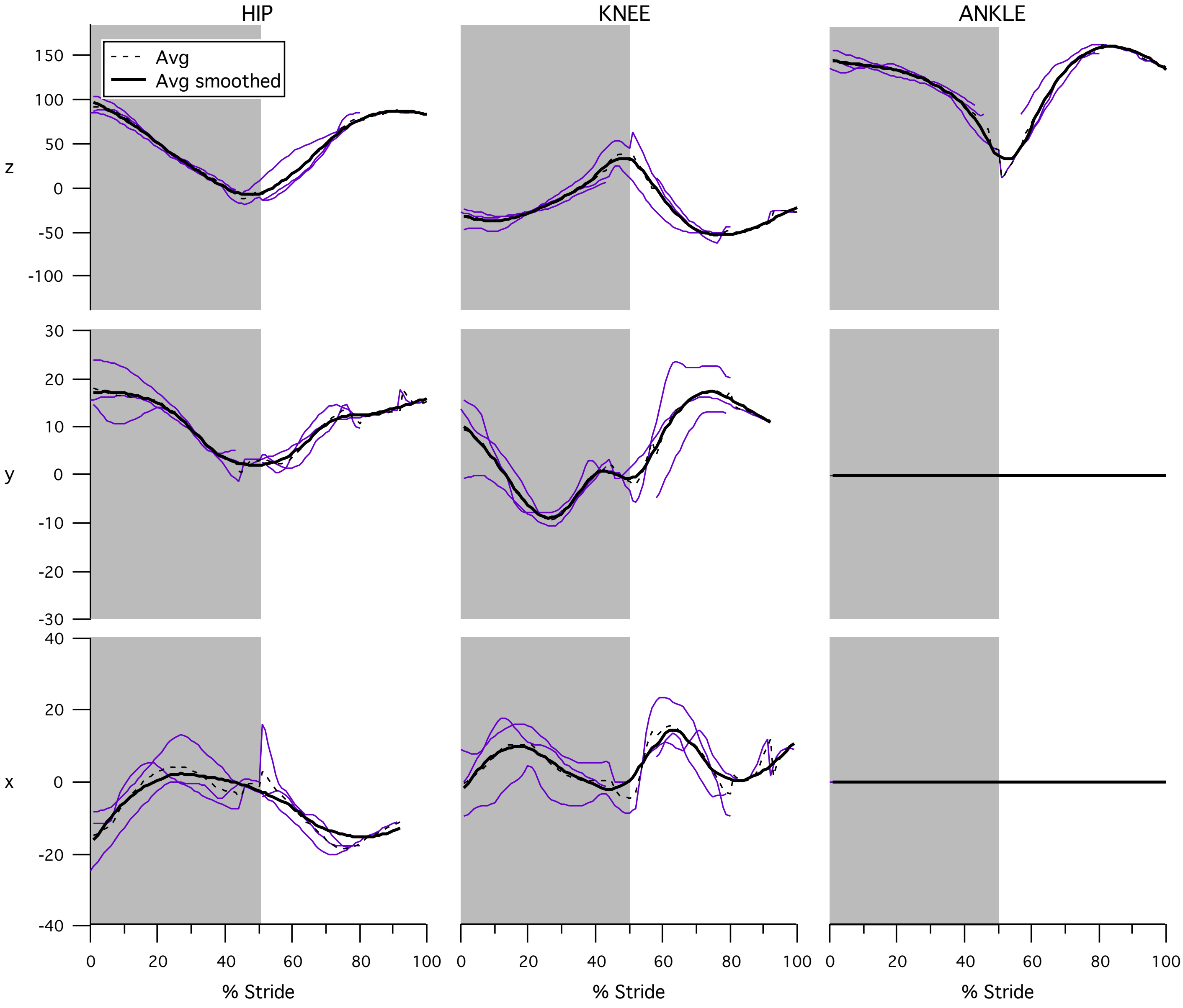

1 bird (cf), three stride cycles; Loess smooth .3-.7
Note that any abrupt changes at stance-swing transitions occur because stance was rotoscoped for one leg of the bird in question, and swing was rotoscoped for the other leg

ONTOGENETIC TRENDS OR DIFFERENCES? (see Table E)
Points are values for individual birds of a given age: 7-8 dph (rank 1), 11-12 dph (2), 18 dph (3), Adult on shallow ramp (4), Adult on steep ramp (5)

Leg Averages
(Stance + Swing)

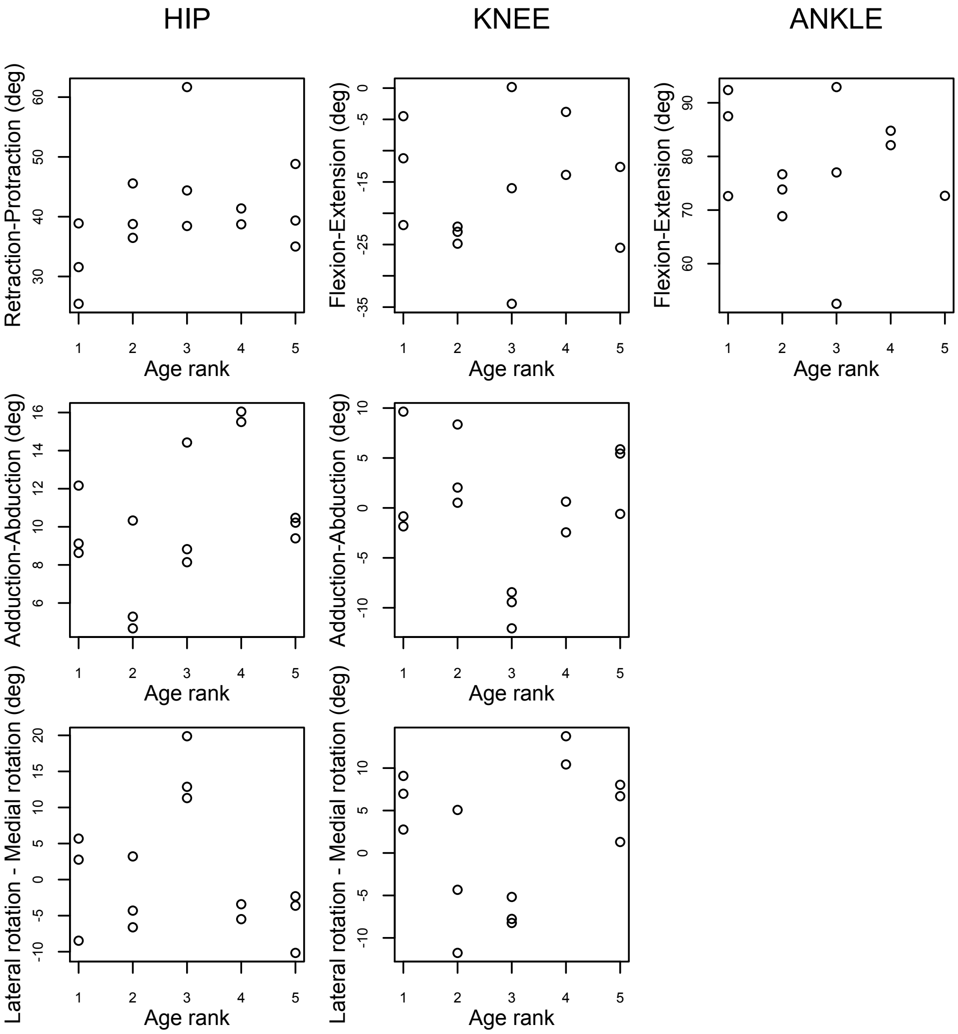

Leg Maxima
(Stance + Swing)

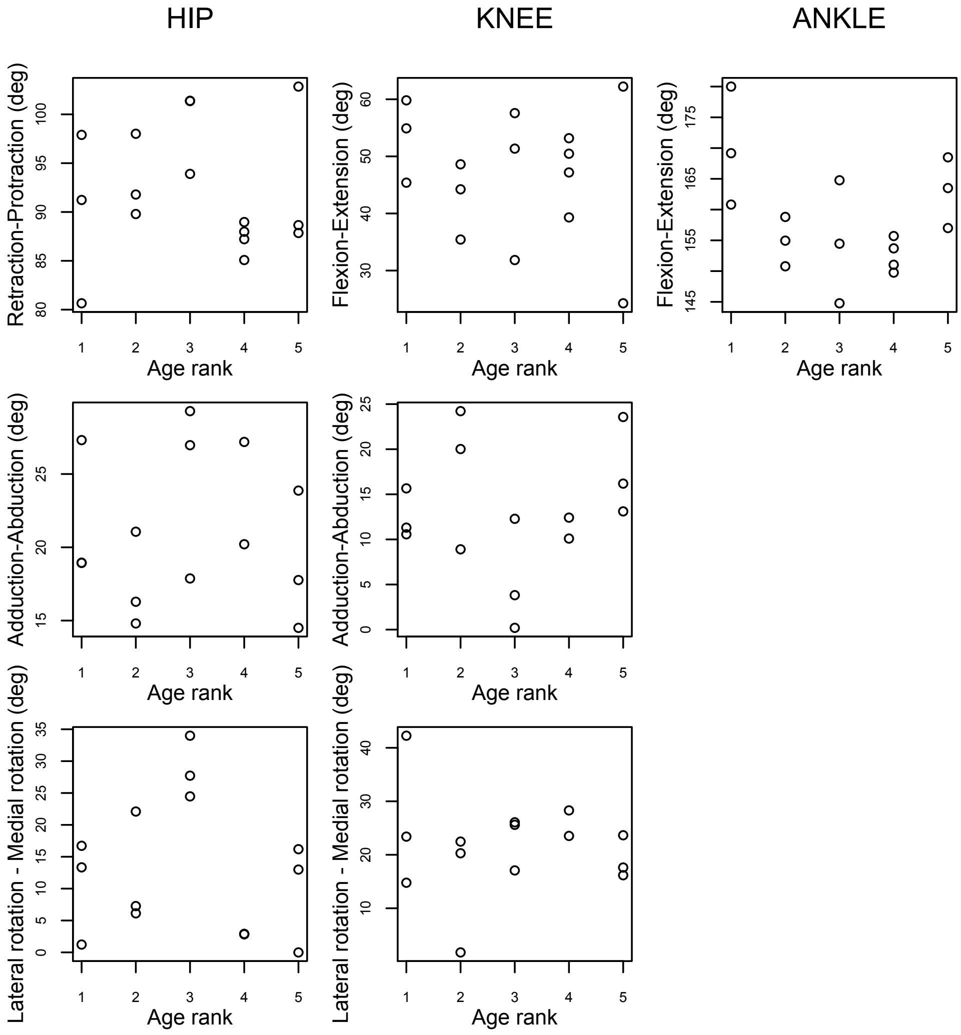

Leg Minima
(Stance + Swing)

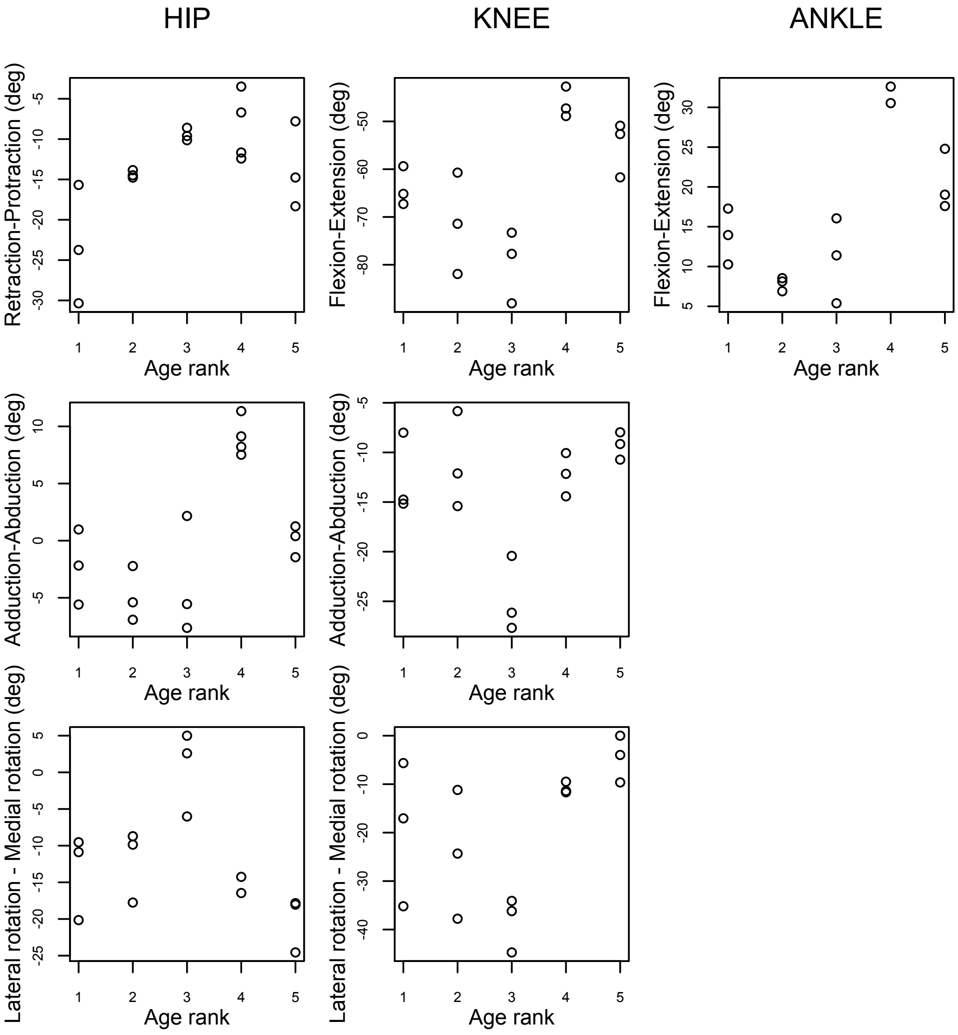

Leg Ranges
(Stance + Swing)

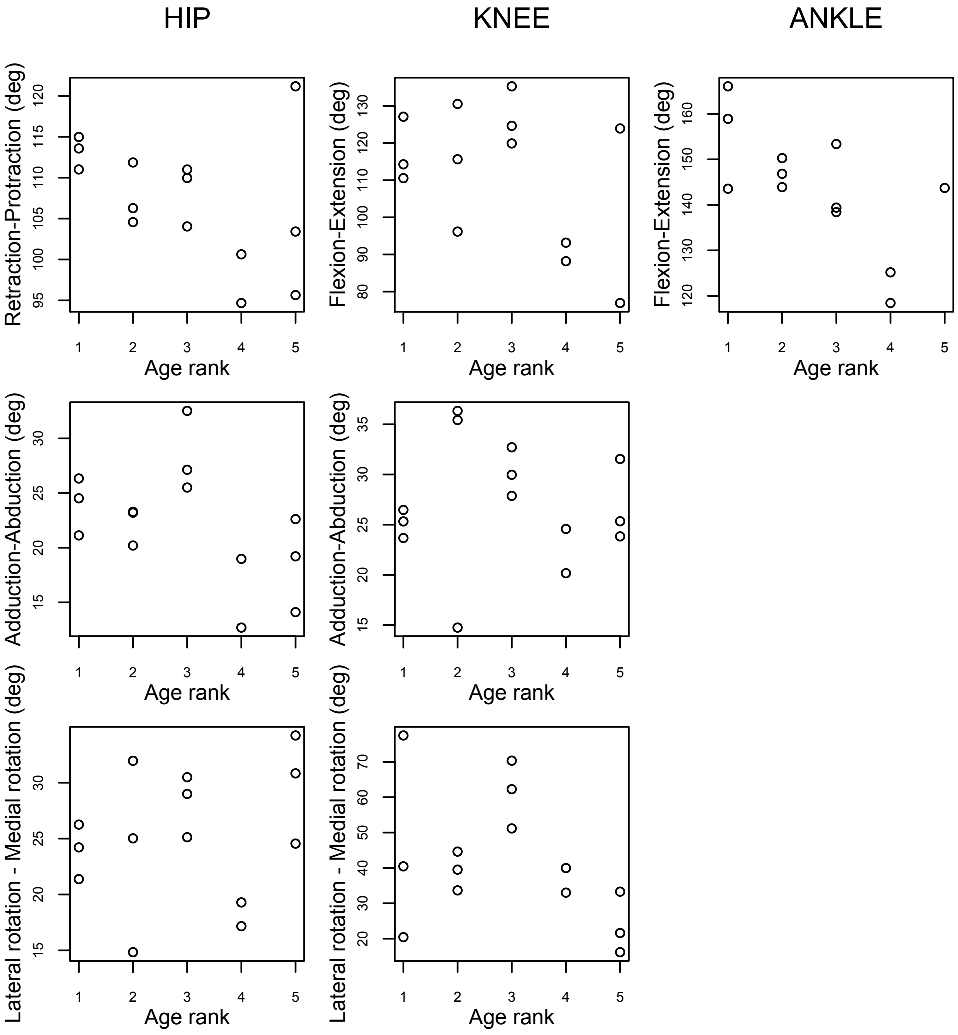

In detail: long axis rotation at Hip

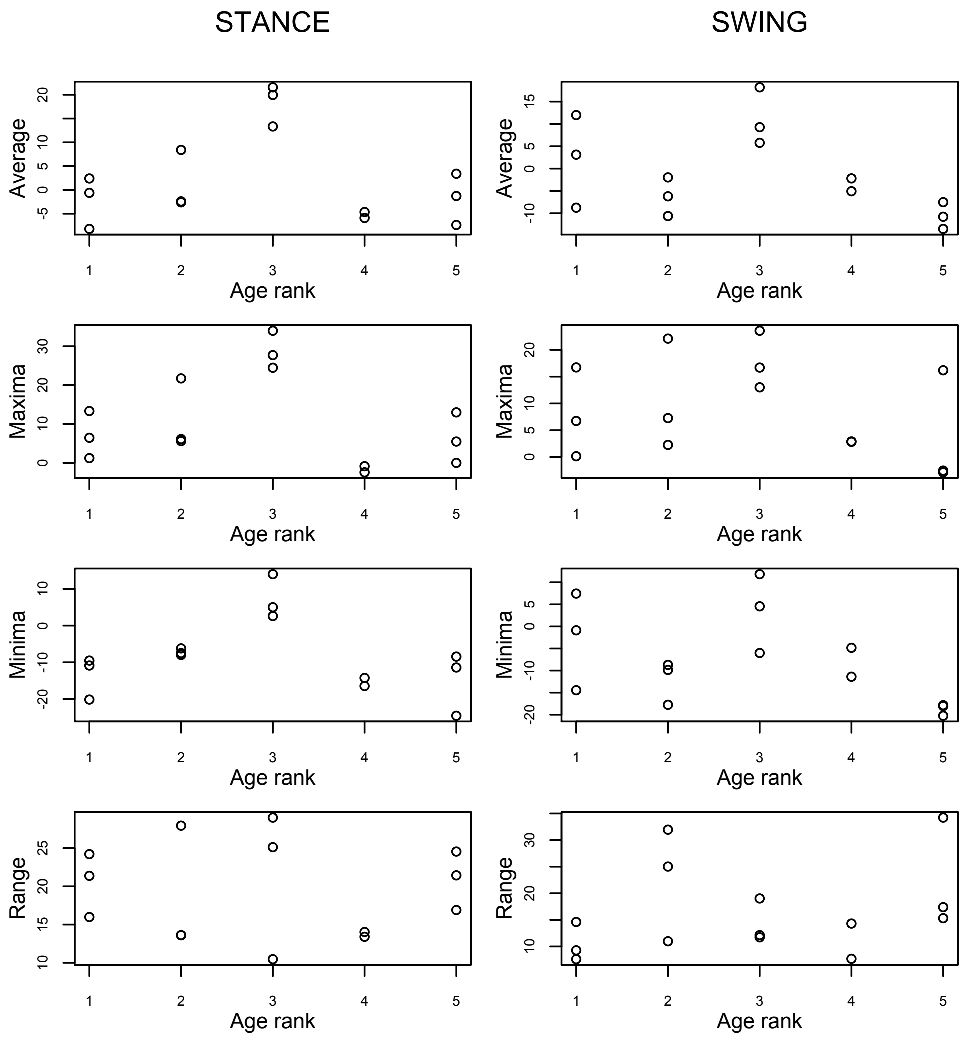

In detail: long axis rotation at Knee

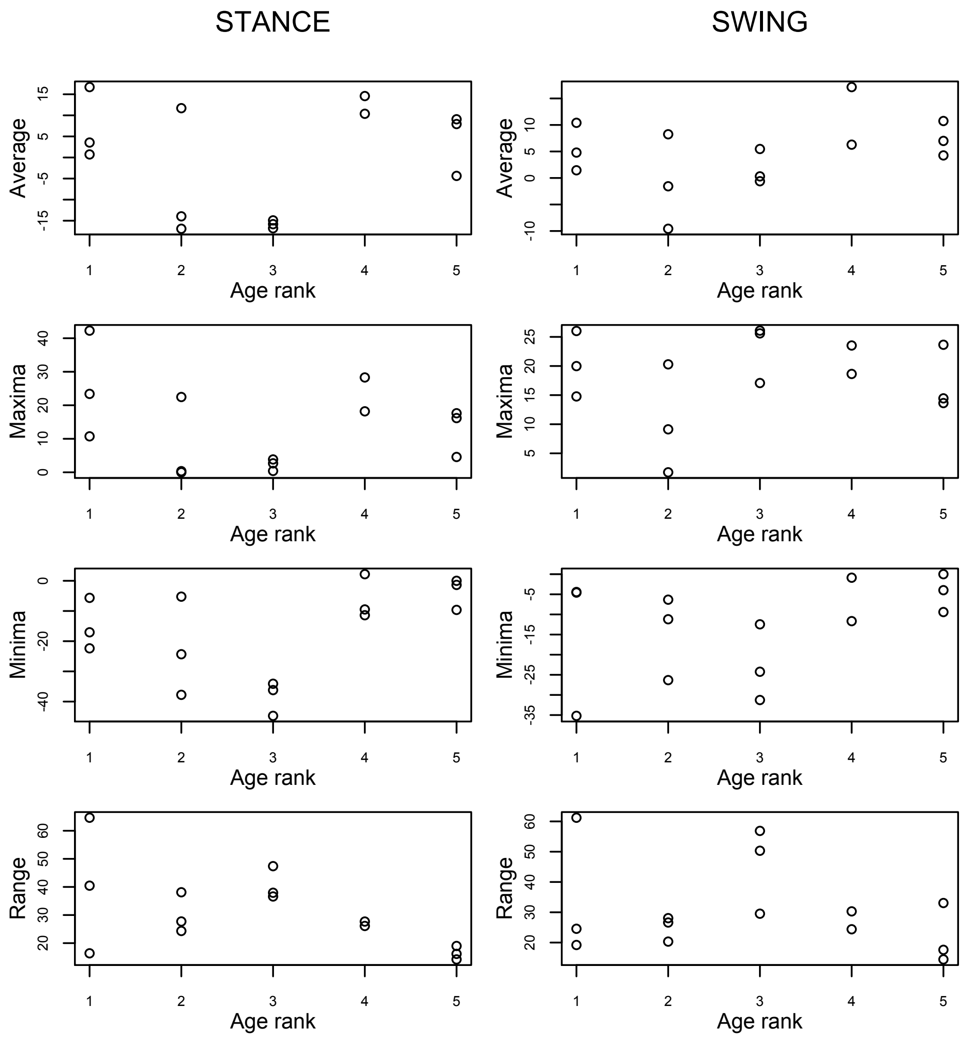

Figure E. Effect of ramp width (H2)

A. Dorsoventral distance between tip of manus and sternum or substrate

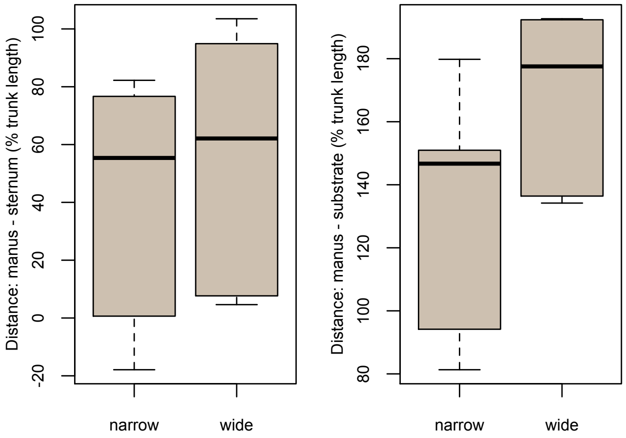

B. Kinematics versus Dorsoventral distance between tip of manus and sternum

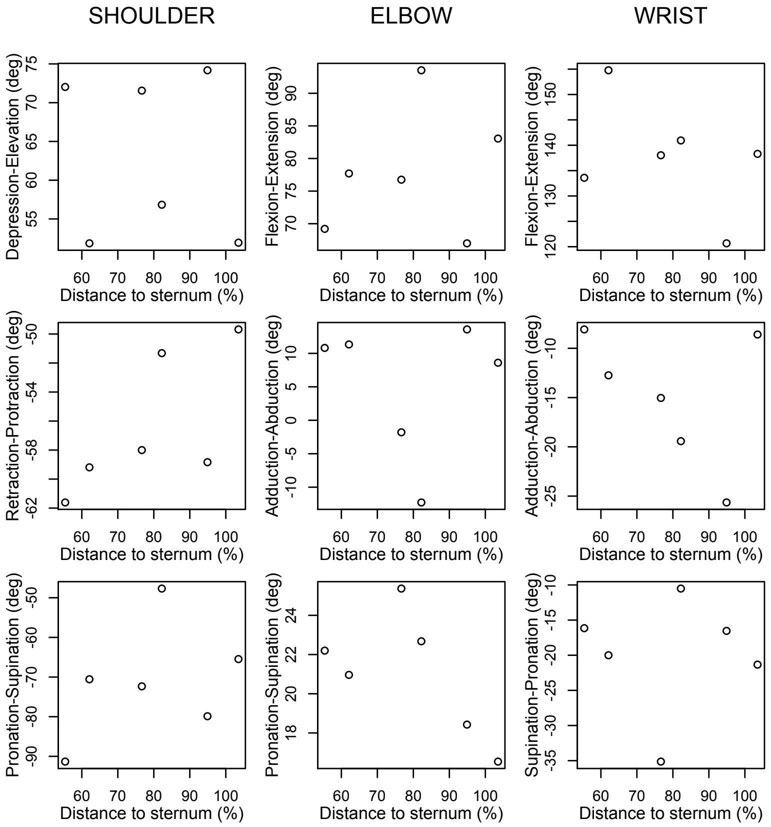

Ramp width does not seem to affect forelimb kinematics. Chukars seem to keep their wings slightly more elevated on wider ramps (A) (Table F), but this does not alter forelimb kinematics (B) (note that trend of decreasing wrist abduction would actually depress, not elevate manus), nor does it prevent birds from depressing their wings more when necessary (i.e., on steeper slopes - Table E).

Figure F. Adults flap-running on shallow (dark purple) **versus steeply** (light purple) **angled ramps**

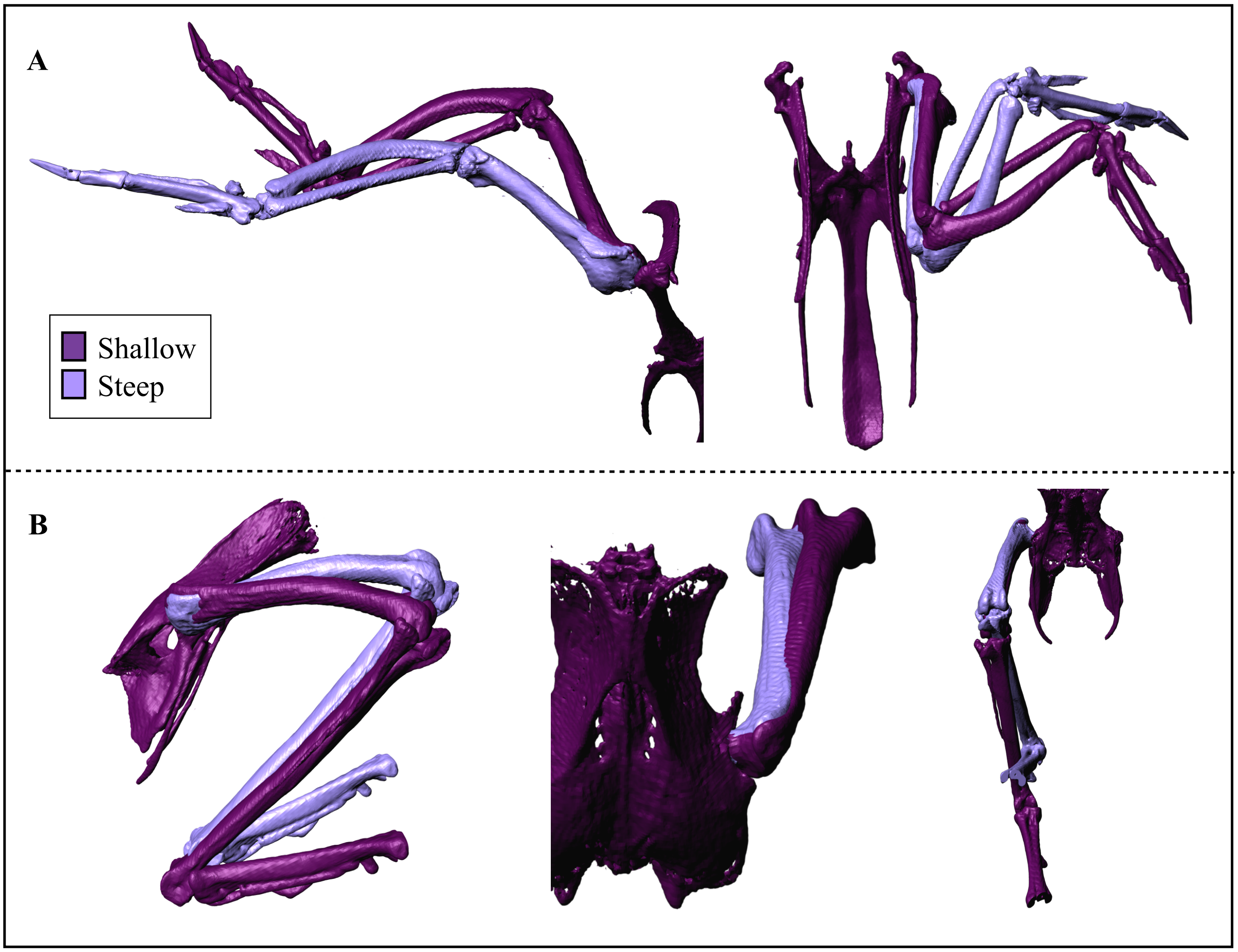

Forelimbs (A). Left: cranial view showing increased depression of the humerus, increased supination of the antebrachium, and increased extension of the wrist of adults at steep angles; late downstroke. Right: dorsal view showing the increased "tucking" motion of adults at steep angles; early upstroke.

Hindlimbs (B). Left: lateral view showing more crouched and more lunge-like movements of adults at steeper angles; late swing. Middle: dorsal view showing reduced lateral splay of adults at steeper angles; late stance. Right: cranial view showing more laterally-directed foot of adults at steeper angles, as well as more crouched and more lunge-like movements; mid-stance.

Figure G. Long leg feathers in extant birds

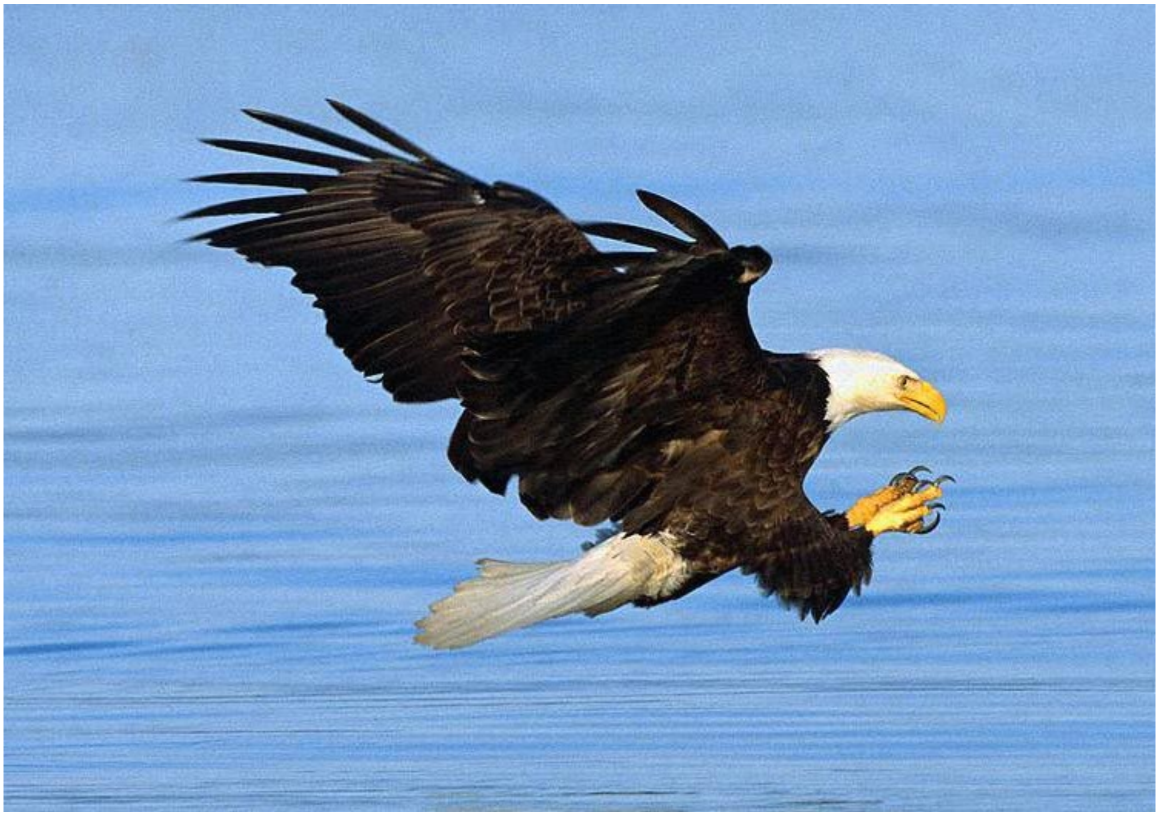

Bald Eagle; free from http://deskpicture.com/

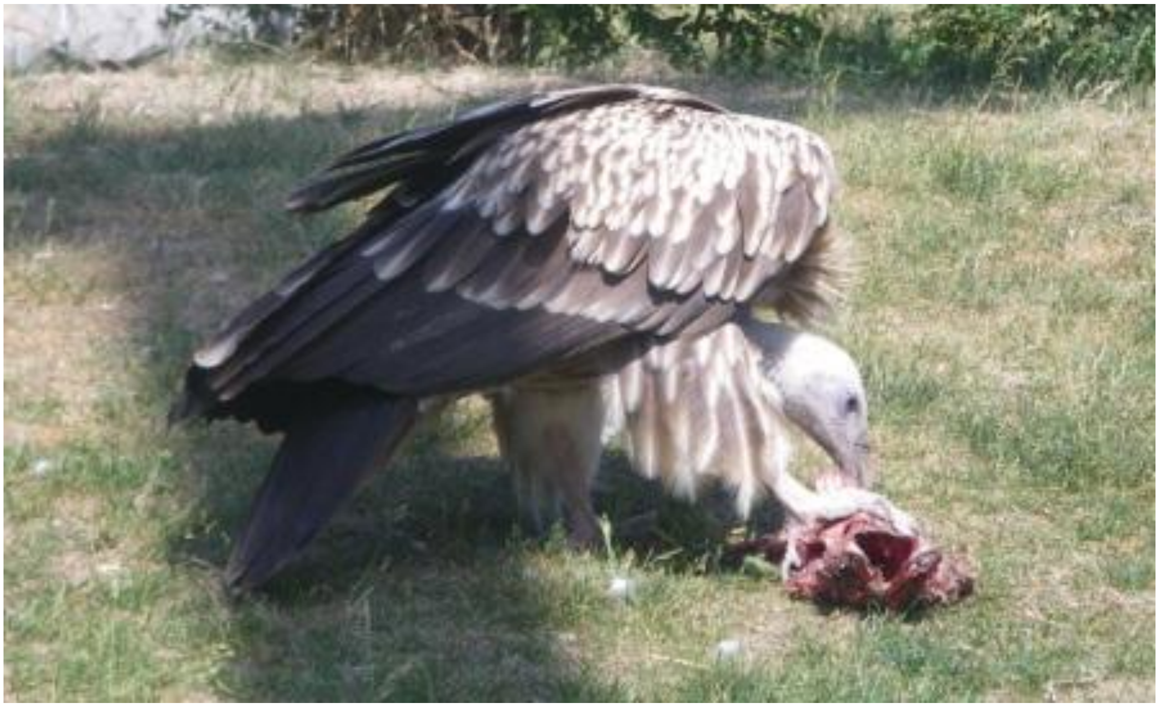

Vulture; reprinted under a CC BY license, with permission from Dr. David Hone, originally posted on https://archosaurmusings.wordpress.com in 2009.

Box A. Washout

Juvenile birds have greater long axis rotation at the elbow and wrist, but both juvenile and adult birds rotate their antebrachia and manuses in opposite directions. This pattern of rotating the antebrachium and manus in opposite directions may achieve washout (lower angles of attack more distally, to avoid stall on the distal wing [9]), though by different mechanisms. Juveniles with small protowings move their wings at relatively low tip velocities [4] that result in little feather deformation; thus juveniles may orient their distal wing at a lower angle of attack by simply by pronating the manus (feathers follow suit). In contrast, adults move their wings at relatively high tip velocities [4], resulting in large amounts of deformation in the primary feathers and a lower angle of attack along the distal wing, such that adults may actually need to counteract some of this feather deformation by supinating the manus. If this is true, then adults achieve washout more passively, via deformation of the primary feathers, whereas juveniles achieve washout more actively, by pronating and supinating the manus throughout stroke cycle.

References

1. Gatesy SM, Baier DB, Jenkins FA, Dial KP. Scientific rotoscoping: a morphology-based method of 3-D motion analysis and visualization. J Exp Zool Part Ecol Genet Physiol. 2010;313A: 244–261. doi:10.1002/jez.588
2. Brainerd EL, Baier DB, Gatesy SM, Hedrick TL, Metzger KA, Gilbert SL, et al. X-ray reconstruction of moving morphology (XROMM): precision, accuracy and applications in comparative biomechanics research. J Exp Zool Part Ecol Genet Physiol. 2010;313A: 262–279. doi:10.1002/jez.589
3. Jackson BE, Segre P, Dial KP. Precocial development of locomotor performance in a ground-dwelling bird (Alectoris chukar): negotiating a three-dimensional terrestrial environment. Proc R Soc B Biol Sci. 2009;276: 3457–3466. doi:10.1098/rspb.2009.0794
4. Heers AM, Tobalske BW, Dial KP. Ontogeny of lift and drag production in ground birds. J Exp Biol. 2011;214: 717–725. doi:10.1242/jeb.051177
5. Heers AM, Dial KP. Wings versus legs in the avian bauplan: Development and evolution of alternative locomotor strategies. Evolution. 2015;69: 305–320. doi:10.1111/evo.12576
6. Dial KP. Wing-Assisted Incline Running and the Evolution of Flight. Science. 2003;299: 402–404. doi:10.1126/science.1078237
7. Heers AM, Dial KP. From extant to extinct: locomotor ontogeny and the evolution of avian flight. Trends Ecol Evol. 2012;27: 296–305. doi:10.1016/j.tree.2011.12.003
8. Baier DB, Gatesy SM, Dial KP. Three-dimensional, high-resolution skeletal kinematics of the avian wing and shoulder during ascending flapping flight and uphill flap-running. PLoS ONE. 2013;8: e63982.
9. Johnson W. Helicopter Theory. United States: Dover Publications Inc.; 1995.
